# Modulating fast skeletal muscle contraction protects skeletal muscle in animal models of Duchenne muscular dystrophy

**DOI:** 10.1172/JCI153837

**Published:** 2023-05-15

**Authors:** Alan J. Russell, Mike DuVall, Ben Barthel, Ying Qian, Angela K. Peter, Breanne L. Newell-Stamper, Kevin Hunt, Sarah Lehman, Molly Madden, Stephen Schlachter, Ben Robertson, Ashleigh Van Deusen, Hector M. Rodriguez, Carlos Vera, Yu Su, Dennis R. Claflin, Susan V. Brooks, Peter Nghiem, Alexis Rutledge, Twlya I. Juehne, Jinsheng Yu, Elisabeth R. Barton, Yangyi E. Luo, Andreas Patsalos, Laszlo Nagy, H. Lee Sweeney, Leslie A. Leinwand, Kevin Koch

**Affiliations:** 1 Edgewise Therapeutics, BioFrontiers Institute, University of Colorado, Boulder, Colorado, USA.; 2 BridgeBio Inc., Palo Alto, California, USA.; 3 Department of Molecular, Cellular, and Developmental Biology and BioFrontiers Institute, University of Colorado, Boulder, Colorado, USA.; 4 Molecular and Integrative Physiology and; 5 Department of Surgery, Section of Plastic Surgery, University of Michigan, Ann Arbor, Michigan, USA.; 6 Department of Veterinary Integrative Biosciences, College of Veterinary Medicine and Biomedical Sciences, Texas A&M University, College Station, Texas, USA.; 7 Genome Technology Access Center, Department of Genetics, Washington University in Saint Louis School of Medicine, Saint Louis, Missouri, USA.; 8 Department of Applied Physiology and Kinesiology and Myology Institute, University of Florida College of Health and Human Performance, Gainesville, Florida, USA.; 9 Departments of Medicine and Biological Chemistry, Johns Hopkins University School of Medicine, Institute for Fundamental Biomedical Research, Johns Hopkins All Children’s Hospital, St. Petersburg, Florida, USA.; 10 Department of Pharmacology and Therapeutics and Myology Institute, University of Florida College of Medicine, Gainesville, Florida, USA.

**Keywords:** Muscle Biology, Therapeutics, Neuromuscular disease, Skeletal muscle

## Abstract

Duchenne muscular dystrophy (DMD) is a lethal muscle disease caused by absence of the protein dystrophin, which acts as a structural link between the basal lamina and contractile machinery to stabilize muscle membranes in response to mechanical stress. In DMD, mechanical stress leads to exaggerated membrane injury and fiber breakdown, with fast fibers being the most susceptible to damage. A major contributor to this injury is muscle contraction, controlled by the motor protein myosin. However, how muscle contraction and fast muscle fiber damage contribute to the pathophysiology of DMD has not been well characterized. We explored the role of fast skeletal muscle contraction in DMD with a potentially novel, selective, orally active inhibitor of fast skeletal muscle myosin, EDG-5506. Surprisingly, even modest decreases of contraction (<15%) were sufficient to protect skeletal muscles in dystrophic *mdx* mice from stress injury. Longer-term treatment also decreased muscle fibrosis in key disease-implicated tissues. Importantly, therapeutic levels of myosin inhibition with EDG-5506 did not detrimentally affect strength or coordination. Finally, in dystrophic dogs, EDG-5506 reversibly reduced circulating muscle injury biomarkers and increased habitual activity. This unexpected biology may represent an important alternative treatment strategy for Duchenne and related myopathies.

## Graphical abstract

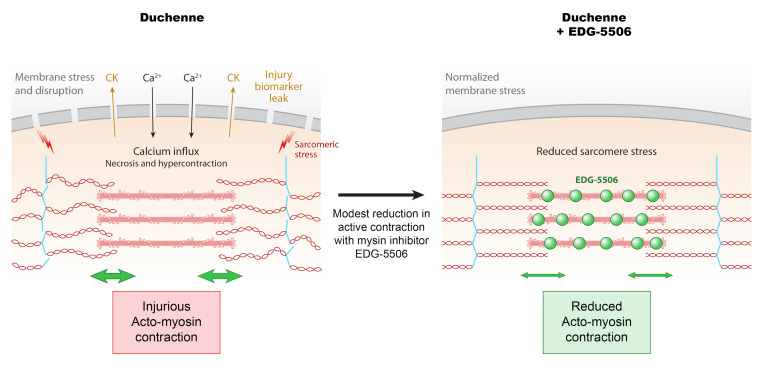

## Introduction

DMD is a lethal, inherited muscle myopathy caused by the absence of dystrophin and destabilization of the dystrophin-glycoprotein complex in the cell membrane (1). Dystrophin provides a structural link between the contractile elements of the sarcomere and the basement membrane of muscle (2). When dystrophin is dysfunctional, mechanical stress (force applied over the muscle area) can lead to the opening of membrane stress channels (such as the TRPC family) and the influx of calcium (3). Ectopic calcium initiates muscle fiber breakdown through several mechanisms, including hypercontraction, activation of proteases, and initiation of mitochondrial apoptotic and necrotic pathways (4). Once muscle fiber breakdown has occurred, skeletal muscle regeneration is possible through the activation of inflammatory pathways and mobilization of muscle stem cells. However, chronic inflammation, cell stress, and stem cell exhaustion reduce the fidelity of this process as patients with DMD get older, leading to fatty and fibrotic tissue accumulation within muscle and compromised physical function (5). Continued muscle degeneration eventually leads to mortality by either cardiac or respiratory muscle failure by the third to fourth decade of life (6).

The connection between mechanical stress and muscle breakdown in DMD has been the subject of scientific research for several decades. Dystrophin is not required for normal sarcomeric organization and contractile activity, as experiments in isolated fibers using dystrophic *mdx* mouse muscle in which membranes have been removed demonstrate normal force and injury resistance. In contrast, contraction of intact *mdx* muscle causes sarcolemmal rupture and force loss (7), particularly under lengthening (eccentric) conditions. Passive lengthening is not sufficient to cause exaggerated injury (8), and removal of contraction by denervation (9) or treatment with high concentrations of the myosin inhibitor, N-benzyl-p-toluene sulfonamide (BTS), also prevents injury (10). The specific nature of structural rearrangements caused by contraction that lead to membrane stress and degeneration in dystrophic muscle is not currently understood.

Adult skeletal muscle consists of two main fiber types, “slow” (type I) and “fast” (types IIa and IIx/d; type IIb is also present in other mammals but not human muscle), defined by the myosin isoform that they express (11). Muscles enriched in fast fibers are more susceptible to mechanical stress in dystrophic mice whereas muscles enriched with slow fibers are more resistant (12). This also appears to be the case in individuals with DMD. Histological studies of young patients with DMD display fiber-type imbalances in the colocalization of fast and embryonic myosin, a marker of regenerating muscle fibers (13). Circulating biomarkers of muscle injury specific to fast but not slow fibers, such as fast troponin I, are also enriched in the plasma of individuals with DMD (14). Histological observations of fast fiber susceptibility have also been made in dog and pig models of DMD (15, 16). With age, slow skeletal muscle fibers also show evidence of injury, including coexpression of embryonic myosin (17). The cause of fast fiber susceptibility to injury and whether it has any influence on slow fiber injury or overall progression of disease is not well understood.

In this study, we used a pharmacological approach to explore the role of muscle fiber contraction in dystrophic muscle stress and degeneration. To achieve this goal, we developed a compound, EDG-5506, which inhibits the ability of myosin to hydrolyze ATP and develop force by decreasing strong binding between myosin and actin within the sarcomere. The high selectivity of this compound for fast but not slow skeletal, cardiac, or smooth muscle allowed us to dissect the role of fast muscle contraction in muscle breakdown and disease progression in models of DMD. Using EDG-5506 ex vivo, in situ and in vivo, we came to the unexpected conclusion that small amounts of fast myosin inhibition result in almost complete protection against skeletal muscle membrane injury, force loss, and longer-term fibrosis. These results point to the possibility that fast myosin inhibition may represent an alternative treatment modality for DMD and other myopathies that are exacerbated by mechanical stress.

## Results

### Contraction via fast skeletal myosin is coupled to force drop and membrane injury in dystrophic muscle.

**Figure 1:**
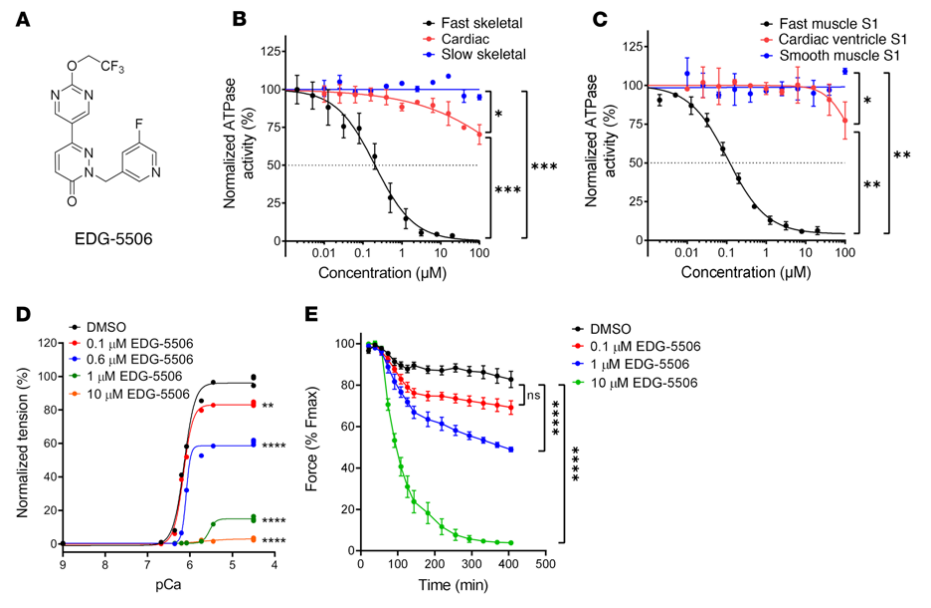
EDG-5506 is a selective inhibitor of fast skeletal myosin ATPase and force generation in fast skeletal muscle. (**A**) Chemical structure of EDG-5506. (**B**) Myofibril ATPase activity curves for EDG-5506, with myofibrils isolated from rabbit fast skeletal muscle, bovine cardiac ventricle, and slow bovine masseter muscle. (**C**) Purified myosin S1 ATPase activity curves for EDG-5506, with rabbit fast skeletal muscle (psoas muscle), pig cardiac muscle, and smooth muscle myosin S1 isolated from chicken gizzard. ATPase activity in the myofibrils is measured at the pCa50 (calcium concentration where ATPase activity is 50% of maximum) value for free calcium for each muscle type (*n* = 2). (**D**) Representative force-calcium curve in single permeabilized fast skeletal muscle fibers (rabbit psoas) with EDG-5506. (**E**) Percentage of initial force with time after addition of EDG-5506 in WT mouse EDL muscle ex vivo. Force was recorded at 250 Hz. Each point represents mean peak force ± 1 SEM (*n* = 4). *P < 0.05; **P < 0.01; ***P < 0.001; ****P < 0.0001. Significance was calculated by 1-way ANOVA with Dunnett’s multiple-comparison test.

To explore the contribution of skeletal muscle contraction to injury in dystrophic muscle, we sought to identify an inhibitor of skeletal contraction that was appropriate for both in vitro and in vivo experiments and was not inhibitory to cardiac or smooth muscle. Contraction of muscle is directly coupled to ATP hydrolysis (18), so we used a high-throughput small-molecule screen measuring fast skeletal muscle myofibril ATPase to identify promising leads (Supplemental Figure 1A; supplemental material available online with this article; https://doi.org/10.1172/JCI153837DS1). Chemical leads were optimized for potency, selectivity, physiochemical and pharmacokinetic properties, leading to the identification of EDG-5506 (Figure 1A). The inhibitory activity and specificity of EDG-5506 was measured using myofibril preparations of different myosin composition. EDG-5506 completely inhibited fast myofibril ATPase from rabbit psoas muscle (96% IIx/d; ref. 19) with an IC_50_ of 0.2 μM but was inactive against cardiac and slow bovine masseter skeletal myofibrils (100% type I) with an IC_50_ greater than 100 μM (Figure 1B). EDG-5506 was also a potent inhibitor of other mixed fast myosin myofibrils (mouse gastrocnemius, IC_50_ 0.4 μM, 21% IIa, 15% IIx, 56% IIb; mouse TA IC_50_ 0.4 μM, mixed IIa, IIx, IIb; ref. 20 and Supplemental Figure 1B), suggesting equal potency against all fast skeletal myosin proteins. In contrast, EDG-5506 was a partial inhibitor of mixed-composition fast/slow human muscle myofibrils (Supplemental Figure 1C, additional selectivity data in Supplemental Table 1). In addition to EDG-5506, we also developed a second myosin inhibitor, EDG-4131 for confirmation of results (for details of the structure and biochemical activity of EDG-4131, see Supplemental Figure 1D and Supplemental Table 2).

The complex nature of these muscle preparations does not allow determination of compound mechanism. Myosin inhibition was confirmed by measuring the actin-activated ATPase of a purified S1 myosin motor subfragment (21). EDG-5506 inhibited the enzymatic activity of fast skeletal S1 from rabbit psoas but not porcine ventricular cardiac S1 or the more unrelated smooth muscle, S1 with an IC_50_ of 0.11 μM and more than 100 μM, respectively (Figure 1C). Other myosin inhibitors, including fast-selective BTS (22) and cardiac-selective mavacamten (23), inhibit inorganic phosphate (Pi) release, the rate-limiting step in the actin-myosin ATPase cycle (Supplemental Figure 1E). We compared the effect of EDG-5506 and BTS on Pi release with actin. Preincubation of EDG-5506 or BTS and myosin (minus nucleotide) for 30 minutes prior to addition of actin decreased the rate of Pi release (Supplemental Figure 1F).

The specificity of EDG-5506 for fast skeletal myosin over other, unrelated proteins was confirmed using two off-target binding and activity screens. EDG-5506 (at 20 μM) did not exhibit any binding affinity with 90 kinase proteins in vitro (KINOMEscan, Eurofins DiscoverX). A second off-target panel (SAFETYscan E/IC_50_ ELECT, Eurofins DiscoverX) tested in vitro enzyme activity against 78 proteins, including GPCR, ion channel, kinases, nuclear hormone receptors, and transporters. In all assays, the IC_50_ of EDG-5506 was more than 10 μM (Supplemental Data File 1).

We next tested the ability of EDG-5506 to reduce force in intact muscle. In detergent-treated single-fiber preparations (24), EDG-5506 selectively reduced force in fast rabbit psoas muscle fibers (Figure 1D) with an IC_50_ of 0.7 μM (Supplemental Figure 1G). In contrast, EDG-5506 had no effect on slow skeletal rat soleus (100% type I) or rat cardiac muscle fibers (Supplemental Figure 1, H and I). In ex vivo assays using mouse extensor digitorum longus (EDL; 100% fast, majority IIb myosin, refs. 20, 25), EDG-5506 reduced force in a concentration- and time-dependent manner, completely inhibiting force at 10 μM (Figure 1E). Submaximal force was also similarly inhibited (Supplemental Figure 1J). Similar experiments with mixed fast/slow mouse soleus (31% type I, 60% type IIa/IIx; ref. 20) muscle yielded partial force inhibition (Supplemental Figure 1K).

**Figure 2:**
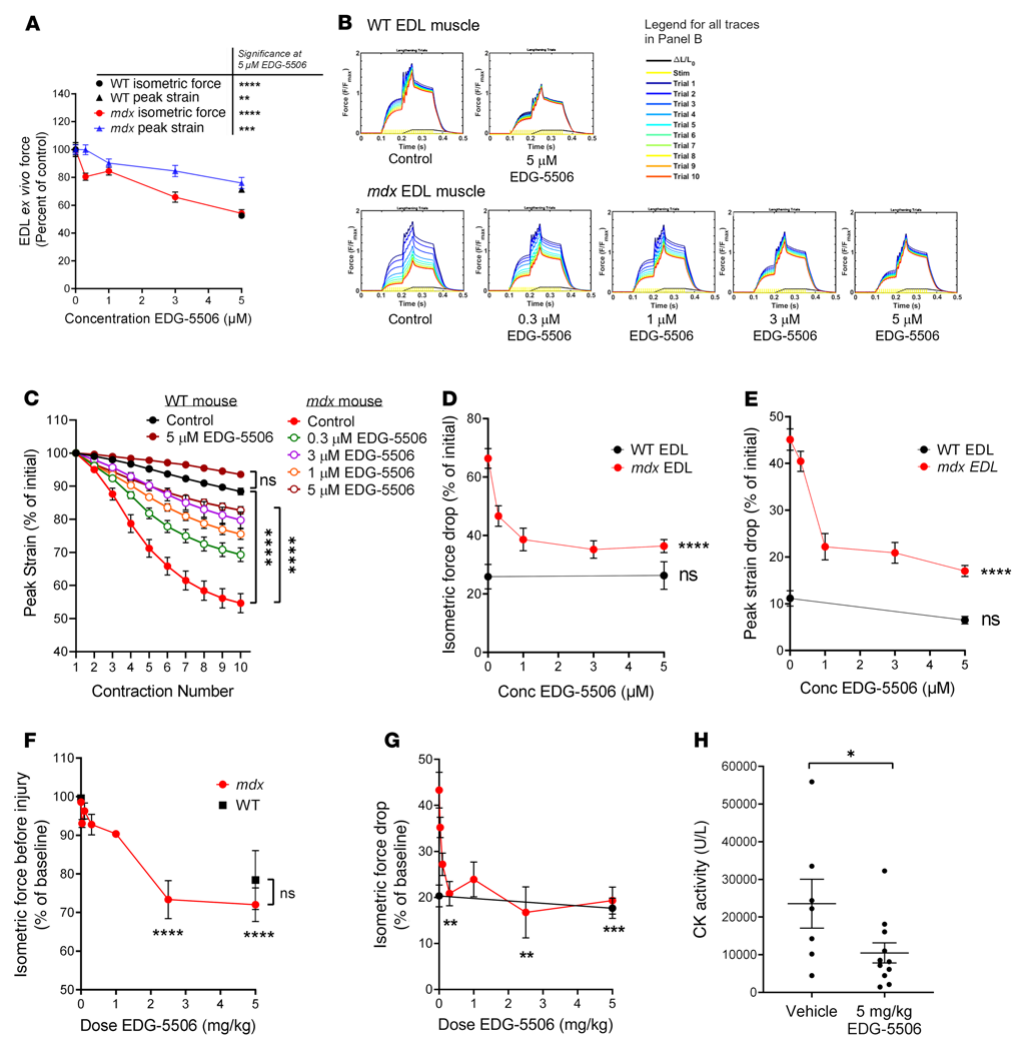
Strength loss during eccentric contraction of dystrophic muscle is dependent upon contraction via myosin. (**A–E**) WT and *mdx* mouse EDL muscle force ex vivo (*n* = 5–14). (**A**) Change in isometric and peak strain as a function of EDG-5506 concentration. Change in isometric force (circles) is represented as a percentage of initial force after 1-hour incubation with EDG-5506. Significance was calculated from the comparison of 5 μM EDG-5506 versus control. Peak strain (triangles) is represented as a percentage of peak strain obtained with vehicle treatment derived from the first eccentric contraction. Definitions of these metrics are provided in Supplemental Figure 2A. (**B**) Example force traces during 10 lengthening contractions of *mdx* and WT mouse EDL muscle ex vivo after incubation with the indicated concentrations of EDG-5506. (**C**) Normalized peak strain with each contraction of the injury protocol (*n* = 4–8). (**D**) Isometric force drop from the first to the last contraction as a function of EDG-5506 concentration. (**E**) Peak strain drop from the first to the last contraction as a function of EDG-5506 concentration. (**F–H**) WT and *mdx* mouse TA muscle force in situ (WT, *n* = 6; *mdx* vehicle, *n* = 17; *mdx* EDG-5506, *n* = 3–5 each). (**F**) Change in isometric force as a function of EDG-5506 dose, represented as a percentage of initial force 3 hours after oral gavage of vehicle or EDG-5506. (**G**) Isometric force drop 10 minutes after 2 lengthening contractions, represented as a percentage of preinjury force. All indicated comparisons were made again using 0 μM EDG-5506 (data not shown). (**H**) CK activity 1 hour after in situ injury (*n* = 7–11). Data are shown as the mean ± SEM. Significance was calculated by 1-way ANOVA with Dunnett’s multiple-comparison test. *P < 0.05; **P < 0.01; ***P < 0.001; ****P < 0.0001. All indicated comparisons were made against results obtained after treatment with vehicle (0 μM EDG-5506).

EDL muscles from *mdx* mice have an exaggerated injury response to lengthening (eccentric) contraction, exhibiting strength loss with repeated contractions (8). EDL muscles were preincubated with EDG-5506, and peak strain and strength loss was measured over 10 rounds of lengthening contractions (Supplemental Figure 2A). EDG-5506 lowered preinjury isometric force and lengthening peak strain in a concentration-dependent manner in both *mdx* and WT muscle (Figure 2A). However, peak strain was inhibited to a lower extent compared with isometric force, leading to an increase in the eccentric/isometric force ratio with EDG-5506 (Supplemental Figure 2B). Strength drop with repeated contractions in *mdx* muscle was dramatically lowered with EDG-5506 (Figure 2, B and C), protecting against isometric and peak force drop (Figure 2, D and E). Both metrics approached but did not achieve WT strength drop, perhaps suggesting other contributing factors. The dose response for protection had a surprisingly nonlinear relationship with near maximal protection at ≤1 μM, a concentration associated with approximately 15% inhibition of isometric force (Figure 2A). Similar protection was also observed with EDG-4131 (Supplemental Figure 2C). Several signaling pathways have been implicated in protection from eccentric injury, including Akt (26), IGF1 (27), and LTBP4/TGF-β (28). To assess whether any of these pathways were directly or indirectly modulated by EDG-5506, EDL muscle from *mdx* mice was incubated with vehicle or 5 μM EDG-5506 for 1 hour prior to analysis with a phosphoprotein array. There were no consistent changes in implicated pathways (Supplemental Data File 2).

We next examined muscle responses in situ, measuring mouse TA muscle force after stimulation of the sciatic nerve with a 2-contraction lengthening injury model (29). Oral administration of EDG-5506 decreased isometric force in a dose-dependent manner (Figure 2F and Supplemental Figure 2D). Lengthening injury yielded an exaggerated strength drop in *mdx* but not WT TA muscle, and it was dramatically improved in *mdx* muscle with EDG-5506 (Figure 2G). Like ex vivo results, protection was achieved at doses associated with small decreases in isometric force (<10%), with force drop similar to that in WT mice. Protection was specific to dystrophic muscle, as there was no decrease in force drop in WT mice (Figure 2G). Unlike ex vivo results, lengthening peak stress was not altered by treatment (Supplemental Figure 2E). EDG-4131 was also protective against in situ injury (Supplemental Figure 2F). Muscle injury is commonly associated with increased circulating creatine kinase (CK). Treatment of *mdx* mice with EDG-5506 prior to in situ injury lowered plasma CK activity compared with that in control mice (Figure 2H). There was no short-term effect of EDG-5506 on CK in the absence of lengthening injury (Supplemental Figure 2G).

**Figure 3:**
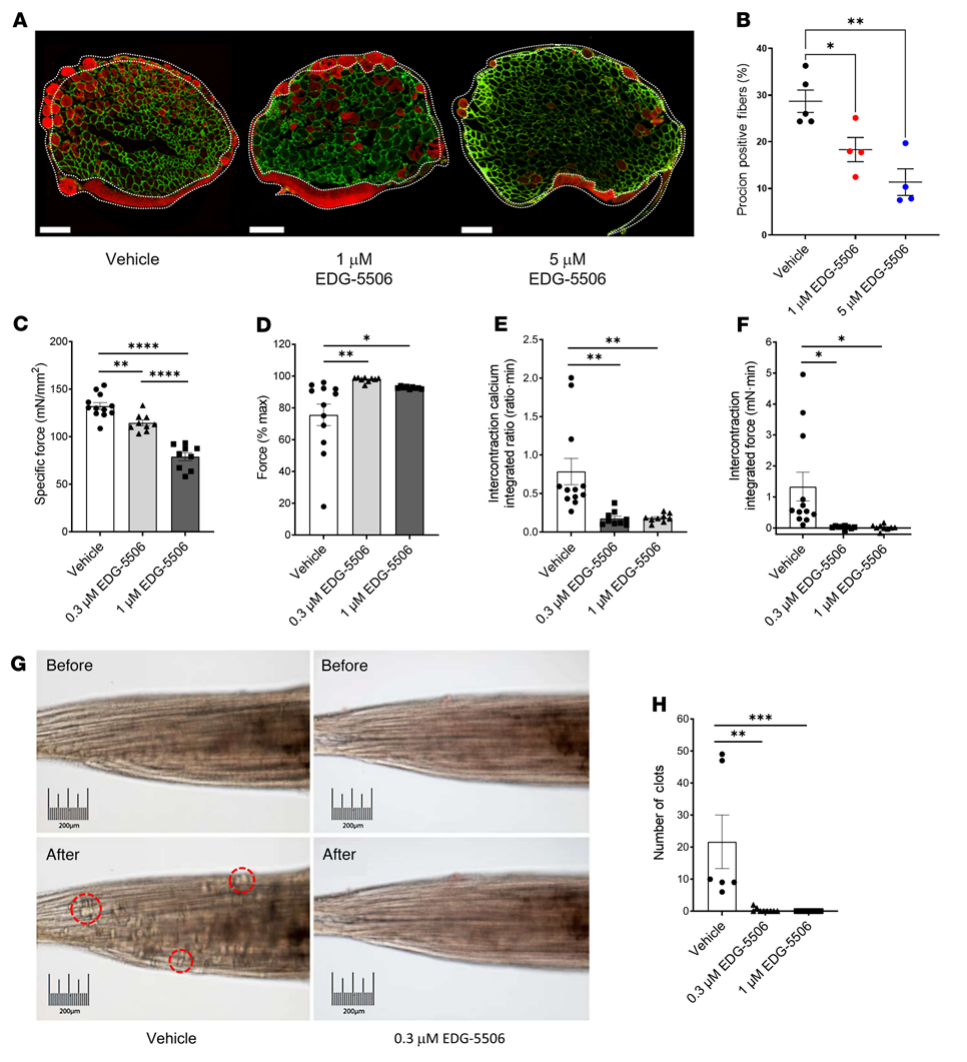
Membrane injury arising from contraction of dystrophic muscle is dependent upon contraction via myosin. (**A**) Representative immunofluorescence images of procion orange-positive fibers after eccentric contraction in *mdx* EDL muscle. Green channel, laminin; red channel, procion orange (*n* = 4). Scale bar: 200 μm. White dotted areas indicate possible nonspecific staining that was excluded during analysis. (**B**) Quantification of procion-positive fibers. (**C**) Specific force prior to injury in *mdx* lumbrical muscles after 1-hour incubation with EDG-5506. (**D**) Force change from the first to the last repeated tetanic contraction of *mdx* lumbrical muscles. (**E**) Average intercontraction fura-2 fluorescence ratio during repeated tetanic contraction. (**F**) Average intercontraction force during repeated tetanic contraction. (**G**) Representative muscle images after 12 contractions. Example clots are highlighted in red. Scale bar: 200 μm. (**H**) Quantification of muscle clots from retracted fibers (*n* = 8–12). Data are shown as the mean ± SEM. Significance was calculated by 1-way ANOVA with Dunnett’s multiple-comparison test. *P < 0.05; **P < 0.01; ***P < 0.001; ****P < 0.0001.

Membrane stress is an important component of eccentric injury in dystrophic muscle (30). We next performed ex vivo lengthening contraction of *mdx* EDL muscle using a membrane-impermeable dye to assess sarcolemmal disruption. EDG-5506 effectively reduced dye uptake (Figure 3, A and B). There was no effect of the dye itself on force (Supplemental Figure 3A). One consequence of membrane disruption is extracellular calcium uptake, a major contributing factor to muscle fiber necrosis (4). We used an ex vivo mouse lumbrical model (31) with fura-2 calcium indicator dye to measure the effect of EDG-5506 on calcium dynamics and uptake with contraction in *mdx* lumbrical muscle. EDG-5506 reduced preinjury force without altering calcium transients (Figure 3C and Supplemental Figure 3B). Contraction caused a strength drop that was completely prevented by EDG-5506 (Figure 3D). EDG-5506 also reduced accumulation of resting calcium and tension (Figure 3, E and F, and Supplemental Figure 3C). Calcium also drives destructive hypercontraction and clotting of fiber myoplasm (31). EDG-5506 completely prevented clot formation (Figure 3, G and H, and Supplemental Videos 1–3). Highlighting the importance of calcium influx to muscle injury (32), we measured a linear relationship between intercontraction calcium and force drop that was corrected with EDG-5506 (Supplemental Figure 3D). Consistent with previous results, maximal efficacy was achieved at 0.3 μM, where specific tension before injury was reduced by 15%.

### EDG-5506 protects skeletal muscle in mdx and DBA/2 mdx mice in vivo.

**Figure 4:**
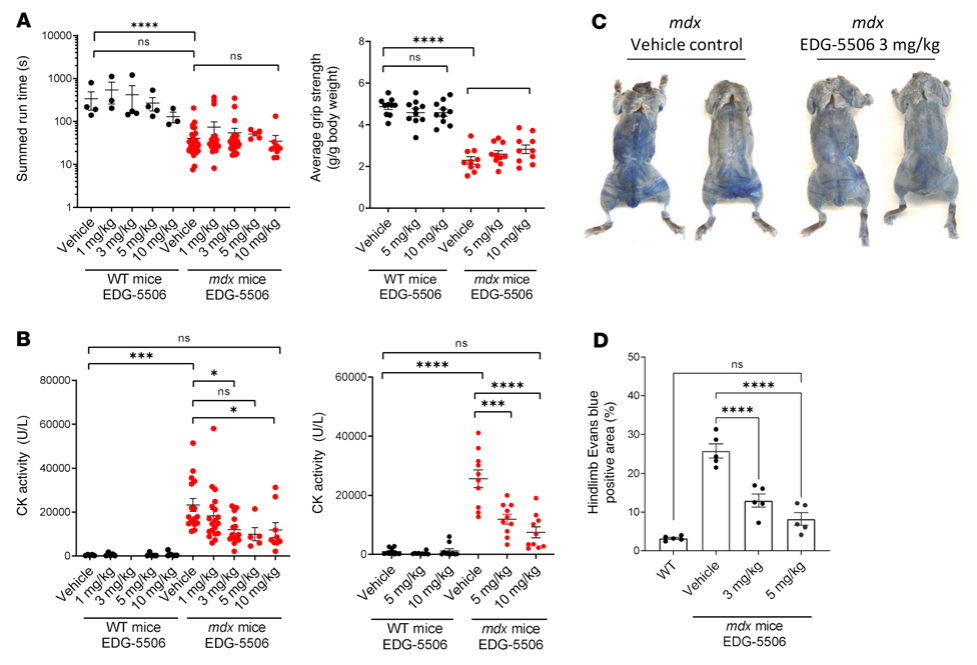
Normalization of membrane permeability with EDG-5506 in *mdx* mice without detrimental effects on strength and coordination in vivo. (**A**) Left: Rotarod performance. Right: Forelimb grip strength 4 hours after oral administration of EDG-5506 (*n* = 10–31). (**B**) Plasma CK activity from blood taken 1 hour after rotarod (left) or grip strength tests (right) (*n* = 10–19). (**C**) Representative whole-body images of nonexercised *mdx* mice 24 hours after intravenous administration of Evans blue dye. Mice were treated for 3 weeks with vehicle or EDG-5506 (these images were reproduced as part of Supplemental Figure 4C). (**D**) Quantitation of Evans blue dye–positive area in the hind limbs of treated mice (*n* = 5) (1–10 mg/kg represents approximately 0.079–0.79 μmol EDG-5506/mouse). Significance was calculated by 1-way ANOVA with Dunnett’s multiple-comparison test. *P < 0.05; ***P < 0.001; ****P < 0.0001.

Mice have more than 90% fast muscle, and its contraction is essential to physical activity (33). We next tested whether protective levels of myosin inhibition impaired strength, coordination, or activity in vivo. Single doses of EDG-5506 up to 10 mg/kg had no effect on grip strength or rotarod endurance in WT or *mdx* mice (Figure 4A). EDG-5506 also had no effect on wheel running (Supplemental Figure 4A). Physical exercise causes enhanced muscle injury in *mdx* mice, particularly with respect to plasma CK (34). We assessed plasma CK after strength and coordination tests. EDG-5506–treated *mdx* mice had significantly lower postexercise CK compared with controls (Figure 4B). There was no effect of single doses of EDG-5506 on CK without exercise (Supplemental Figure 4B). Membrane permeability can also be visualized in vivo via Evans blue dye (EBD) muscle uptake (35). Three-week treatment of *mdx* mice with EDG-5506 reduced blue fibers close to the level seen in WT mice, demonstrating muscle protection with longer compound treatment without formal exercise (Figure 4, C and D, and Supplemental Figure 4C).

**Figure 5:**
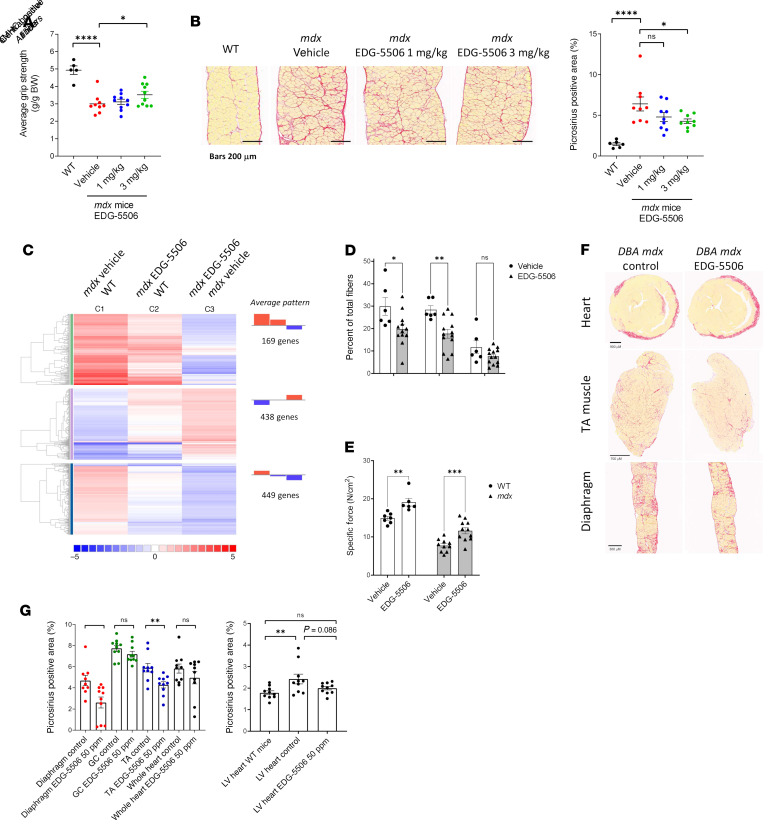
Longer-term exposure of protective levels of myosin inhibition are sufficient to decrease muscle degeneration and fibrosis in *mdx* mice. (**A**) Average grip strength (experimenter blinded) measured after 5 weeks of dosing in *mdx* mice (*n* = 5–10). (**B**) Left: Representative images. Right: Quantification of collagen (stained with picrosirius red) in *mdx* mouse diaphragm after 8 weeks of treatment (*n* = 9–10). Scale bar: 200 μm. (**C**) RNA-Seq meta-analysis. Colors are graded by log_2_ fold change (WT, *n* = 2; *mdx* vehicle, EDG-5506, *n* = 3). (**D**) Histological quantification of central nuclei and eMHC-positive fibers in soleus muscle sections from post-weaning *mdx* mice after 3 weeks EDG-5506 administration. (**E**) Specific force in the soleus muscle ex vivo in postweaning *mdx* and WT mice after 3 weeks of treatment with EDG-5506 or vehicle. (**F**) Representative histology sections examining muscle fibrosis in DBA/2 *mdx* mice after 12 weeks of treatment with control or EDG-5506 chow (50 ppm or 0.13 mmol/Kg chow). Scale bar: 900 μm (heart); 700 μm (anterior tibialis [TA] muscle); 300 μm (diaphragm). (**G**) Quantification of collagen (picrosirius red area). Left: Collagen quantification in select muscles (GC, gastrocnemius). Right: Collagen quantification in the left ventricle (LV; *n* = 9–10). Data are shown as the mean ± SEM. Significance was calculated by 1-way ANOVA with Dunnett’s multiple-comparison test. *P < 0.05; **P < 0.01; ***P < 0.001; ****P < 0.0001.

Muscle degeneration in *mdx* mice leads to fibrosis, particularly in the diaphragm (36). We next tested whether chronic treatment with EDG-5506 would benefit fibrosis in *mdx* mice. Seven-week-old *mdx* mice were dosed with EDG-5506 for 8 weeks. EDG-5506 increased average grip strength (Figure 5A) and decreased diaphragm fibrosis compared with placebo (Figure 5B) and returned the skeletal muscle transcription profile closer to that of WT mice (Figure 5C and Supplemental Data File 3). EDG-5506 had no effect on body or muscle weight, and there was a trend toward fewer small, fragmented fibers in the TA muscle but not the diaphragm (Supplemental Figure 5, A–D). A rotarod test administered midstudy confirmed sustained membrane protection with decreased exercise-associated plasma CK (Supplemental Figure 5E). To better understand the effect of EDG-5506 on muscle degeneration, we performed a second study in young *mdx* mice before muscle necrosis that peaked at weaning (3–4 weeks), with the soleus muscle particularly affected (37). EDG-5506 decreased central nucleation, with a trend toward decreased embryonic myosin-positive fibers in the soleus (Figure 5D). Specific tension was also higher in the soleus of treated WT and *mdx* mice (Figure 5E). Muscle size, fiber size, and proportion of fast (IIA) fibers was unaffected (Supplemental Figure 5, F–H).

Unlike patients with DMD, *mdx* mice exhibit limited muscle fibrosis (36). Therefore, we performed studies in the more fibrotic DBA/2J *mdx* mouse (38). Beyond fibrosis, DBA/2J *mdx* mice exhibit more fragmented, smaller fibers (39). EDG-5506 had no effect on body or muscle weights, but the diaphragm muscle had fewer small fibers after 12 weeks of dosing (Supplemental Figure 5, I–K). Treatment also reduced fibrosis in TA and diaphragm muscles, with a trend toward lower fibrosis in the left ventricle (Figure 5, F and G). Studies in DBA/2J *mdx* mice with EDG-4131 confirmed this pattern, including significant decreases in left ventricular fibrosis (Supplemental Figure 5, I–M).

### EDG-5506 lowers disease biomarkers and improves physical function in DMD dogs.

**Figure 6:**
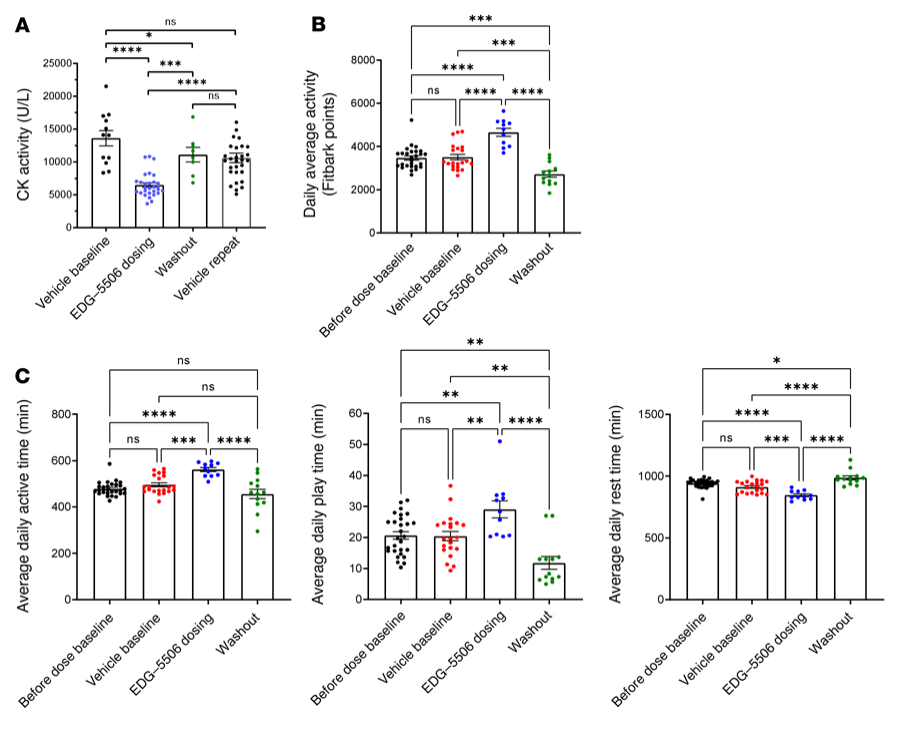
Selective inhibition of active contraction in fast skeletal muscle decreases CK and increases habitual activity in DMD dogs. (**A**) Plasma CK activity in 7-month-old DMD dogs (*n* = 4) before, during, and after 14 days oral gavage with EDG-5506. Each data point represents CK activity from an individual blood draw (2–3 draws during the vehicle baseline, 5–8 draws during the dosing period, and 2 draws during the vehicle washout). In a separate study, the same 4 dogs were dosed daily with vehicle, and blood CK was at regular intervals for 14 days (indicated as vehicle repeat, 6–9 blood draws per dog). Data are shown as the mean ± SEM. (**B**) Average daily activity measures from an electronic activity monitor (FitBark) in the same DMD dogs (*n* = 3, 15 months old) before, during, and after an 11-day oral gavage with EDG-5506 (2 mg/kg daily for 4 days, then every other day). Each data point represents the daily average activity for the 3 dogs. (**C**) Timed function data from the same activity monitors. Average daily active time, average daily play time, and average daily rest time. Each point represents average activity for 1 day (2 mg/kg represents approximately 68.2 μmol EDG-5506/dog). Significance was calculated by 1-way ANOVA with Tukey’s multiple-comparison correction. *P < 0.05; **P < 0.01; ***P < 0.001; ****P < 0.0001.

We viewed data in mice as encouraging but were interested in testing EDG-5506 in dystrophic golden retriever (GRMD) dogs; their muscle composition and function more closely resemble that of humans (40). Older (7 months of age), disease-stable GRMD were dosed in 2-week periods with vehicle, then EDG-5506, followed by a vehicle washout. As fast fiber atrophy has been documented in DMD dogs (16), we confirmed sustained fast myosin from biopsies of age-matched WT/GRMD gastrocnemius muscle (Supplemental Figure 6A). EDG-5506 concentration was measured from a muscle biopsy at the end of the dosing period (4,951 ± 217 ng/g muscle). EDG-5506 administration was associated with a more than 50% decrease in CK, returning to pretreatment levels after compound washout (Figure 6A). Physical activity normally increases CK in GRMD (41), raising the question of whether EDG-5506 lowered CK via decreasing physical activity. We sought to test the effect of EDG-5506 on habitual activity in the same dogs using an activity monitor (42). EDG-5506 improved average daily activity by more than 30% (Figure 6B and Supplemental Figure 6B) with reversible increases in time active and decreases in time resting (Figure 6C). One GRMD used in the biomarker study was too severely progressed to participate in this study. Activity in this dog did not change over the same dosing period (Supplemental Figure 6C*)*.

**Figure 7:**
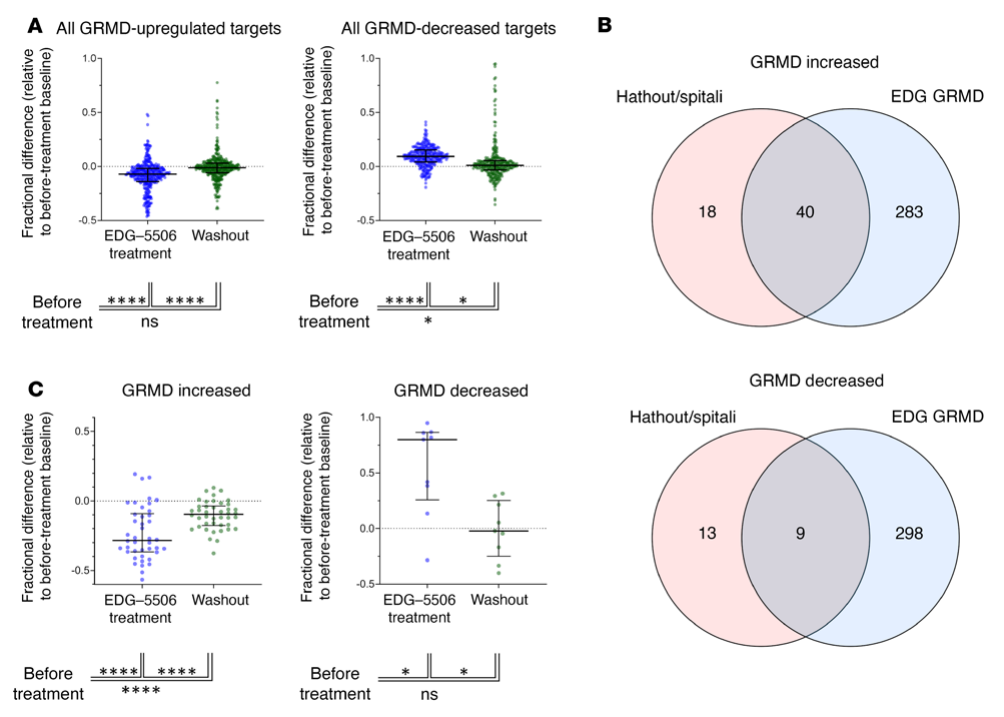
Selective inhibition of contraction in fast skeletal muscle reverses proteomic signatures associated with disease in DMD dogs. (**A**) Effect of EDG-5506 treatment on plasma proteins identified by Somascan as increased (left) or decreased (right) in DMD dogs compared with healthy littermates. Fractional change was then calculated for each target during the treatment and washout period, relative to the predose baseline. Data are shown as the median ± interquartile range. (**B**) Overlap of GRMD increased and decreased proteins with those from a common data set from a patient with DMD (43). (**C**) Effect of treatment with EDG-5506 on common DMD-elevated (left) or -reduced (right) proteins. Data are shown as the median ± interquartile range. See Supplemental Data File 3 for full analysis. Significance was calculated by 1-way ANOVA with Tukey’s multiple-comparison correction. *P < 0.05; **P < 0.01; ***P < 0.001; ****P < 0.0001.

Somascan plasma proteomics has been used in patients with DMD to generate a distinguishing protein signature compared with healthy controls (43). As CK was decreased with EDG-5506, we asked whether other proteins associated with DMD were also changed. Somascan comparison of GRMD plasma with healthy dog plasma revealed many protein differences (Supplemental Data File 4). We examined the effect of EDG-5506 on the proteomic signature of GRMD. Treatment reversibly lowered GRMD-elevated proteins and increased GRMD-downregulated proteins (Figure 7A and Supplemental Data File 5). Gene ontology term analysis revealed decreases in pathways associated with dystrophic muscle, including apoptotic, cellular signaling, metabolic, and immune responses (Supplemental Table 3). Comparison of the GRMD proteomic signature with that from DMD (43) revealed a common set of 40 elevated and 9 depleted proteins (Figure 7B). EDG-5506 also significantly reversed this common DMD signature (Figure 7C and Supplemental Data File 6).

## Discussion

These studies reveal a surprising sensitivity of dystrophic muscle to sarcomeric and membrane stabilization by inhibition of force by fast skeletal muscle myosin. Following the experiments reported here, reports have also proposed fast myosin inhibition as a strategy for muscle relaxation in myotonia or spasticity (44). Our findings uncover a connection between muscle contraction and dystrophic muscle injury and expand the possible uses for this class of compounds into DMD, a progressive, grave disease. This relationship may also apply to other myopathies where muscle contraction causes fiber breakdown, including types of limb girdle muscular dystrophy, Becker muscular dystrophy, and metabolic myopathies such as McArdle’s disease (45).

Protective effects from myosin inhibition build on earlier reports of dystrophic muscle protection by ablation of muscle contraction by unloading, denervation, or immobilization with the myosin inhibitor BTS (10). In contrast to these approaches, we demonstrate that full immobilization is not required for protection and that modest force inhibition (10%–15%) is sufficient to prevent dystrophic muscle injury. Our initial assumption was that muscle protection via this mechanism would occur through decreased contraction peak strain, a key contributor to injury in dystrophic muscle (8, 30). The results of the present study are inconsistent with this hypothesis, as EDG-5506 had only modest effects on peak strain ex vivo and no effect in situ. Examination of the ratio between isometric force and peak strain, another factor influencing dystrophic muscle injury (8), revealed an increase with treatment (Supplemental Figure 2C). This is consistent with reports showing decreased isometric force but increased force enhancement with lengthening in skeletal muscle fibers treated with myosin inhibitors such as BDM and vanadate (46, 47). Mechanistically, it has been proposed that inhibitors that increase the weakly bound myosin ADP.Pi state convert to a strongly bound state with lengthening. Functional consequences of this lengthening enhancement are unclear.

The magnitude of eccentric stress in our injury models is not commonly seen in sedentary mice, yet we measured significant decreases in EBD incorporation with EDG-5506. A hypothesis for protection in sedentary mice is based on an established model (31) where muscle fiber stress causes localized increases in calcium and nonuniform contraction of serial sarcomeres. In dystrophic muscle, the lack of costameres amplifies stress along serial sarcomeres, resulting in further membrane stress and calcium entry. Under these conditions, EDG-5506 would provide protection against local stress by limiting uneven contraction.

A caveat with any small-molecule approach is that off-target activities might contribute to positive pharmacology. Documented pathways that also confer protective effects in dystrophic muscle include sarcolemmal nNOS (48) and membrane excitability (49). Consistency between ex vivo (where blood supply is severed) and in situ results with EDG-5506 suggest that blood perfusion and NOS activity are not essential for protection. Similarly, decreases in force but not calcium in treated lumbrical muscles suggest EDG-5506 does not decrease membrane excitability. Additional studies also explored the promiscuity of EDG-5506 via in vitro binding, enzymatic, and phospho-antibody arrays, confirming low off-target activity for EDG-5506. Regardless, there is still a possibility that other unknown activities of EDG-5506 might contribute to its protective effects.

Protective doses of EDG-5506 had no negative effects on strength, coordination, or habitual activity in mice, suggesting functional accommodation or excess capacity is present in skeletal muscle. Mouse muscles are composed of primarily fast fibers while human muscles are composed of an approximately even split of slow and fast fibers (50). Muscle contraction is controlled by both motor neuron stimulation rate and coordinated recruitment of populations (units) of fast or slow fibers (51). As physical demands increase, stimulation rates and recruitment increases with slow units usually recruiting before faster units (51). This system is designed to maintain physical performance even under extreme stress. Inhibition of fast myosin via EDG-5506 would be expected to reduce fast fiber force but overall muscle performance might still be maintained via force adjustment provided by stimulation and recruitment of motor units. Genetic evidence for both accommodation and adverse effects from total fast fiber inhibition exists in individuals with null mutations in MYH2, the gene encoding myosin IIa, which makes up 85% of human fast fibers (52). MHY2-null skeletal muscles have few fast fibers, but individuals remain ambulatory although they manifest mild-to-moderate proximal limb and oculofacial weakness and dysfunctional extraocular muscles.

Although selective injury of fast skeletal muscle fibers in DMD was first documented over 30 years ago (13), its cause and relevance to disease progression is poorly understood. Fast fibers differ in many ways from slow fibers that might heighten susceptibility. Possible factors include lower levels of the dystrophin-related protein utrophin (53), lower oxidative and mitochondrial capacity, and differences in organization of the z-disc, making it more susceptible to disruption (54). Muscle injury studies in healthy volunteers have shown elevation of circulating troponin I from fast but not slow muscle (55), suggesting that fast fiber sensitivity is perhaps normal physiology amplified in the absence of dystrophin.

Older patients with DMD (≥7 years) exhibit muscle fiber dysfunction in both slow and fast fibers, including expression of embryonic myosin (17). It is unclear whether fast fiber damage indirectly affects slow fibers or whether injury occurs directly over a longer time. One nuance of the biochemical profile of EDG-5506 is that it inhibits both fast and embryonic myosin (Supplemental Table 1). Embryonic myosin, present with fiber regeneration, is present in approximately 30% of fibers from patients with DMD (56) and should be targeted by EDG-5506, whether they coexpress fast or slow myosin. As embryonic myosin is expressed during muscle regeneration, we confirmed that EDG-5506 did not interfere with muscle repair using a cardiotoxin model. A surprising finding of this study was that muscle size was larger with EDG-5506 treatment (Supplemental Figure 7). Young *mdx* mice undergo large-scale muscle degeneration/regeneration at 3–4 weeks of age (37). EDG-5506 was protective against this degeneration, reducing central nuclei. While neither muscle nor fiber size was increased in this study, specific tension occurred in both *mdx* and WT mice and was higher in these animals than in controls (that exhibit no degeneration). Why myosin inhibition would increase either muscle size or function in these dynamic situations is not currently understood.

Our results in older GRMD help understanding of compound response in older patients with DMD. GRMD exhibit early disease onset that stabilizes at 6 months. Results from studies of 7- to 18-month-old GRMD can be compared with those of patients older than 10 years with DMD, with muscle weakness and increased occurrence of mixed composition fast/slow fibers (40). In this light, it is encouraging that we observed both decreased CK and increased activity with EDG-5506. There are few precedents for acute functional improvements in GRMD. The closest exemplar in DMD dogs would be glucocorticoids, which increased some aspects of physical function and lowered CK (in combination with cyclosporine) but had longer-term adverse effects, including muscle atrophy and calcification (57).

Our use of Somascan proteomics on samples from GRMD facilitated a detailed analysis of disease-associated biomarkers in this model. One caveat to these data is that Somascan uses aptamers for human proteins with unknown cross-reactivity against dog. Given this, we saw consistent patterns between GRMD and DMD signatures with overlap between published DMD signatures (43), further validating GRMD as a close comparator to DMD. Treatment with EDG-5506 reversed this overlapping signature, speaking to a universal proteomic correction of disease in GRMD and, by extension, DMD. Limitations of this study include in vivo differences between *mdx* mice, GRMD, and patients with DMD, and the short duration and lack of force measures in our GRMD study.

Our studies demonstrating common beneficial effects of EDG-5506 across preclinical models are unusual and speak to its fundamental nature. EDG-5506 protects muscle ex vivo and in situ from contraction injury with a response that compares favorably to AAV microdystrophin replacement strategies (58, 59). Protection also extended to the heart, with lower cardiac fibrosis in DBA/2J *mdx* mice, a finding distinct from AAV microdystrophin in the same model (60). Skeletal muscle rescue can improve cardiac health in *mdx* and double-knockout dys/utr mice (61). Additional studies will be required to understand cardiac protection with EDG-5506.

In summary, these findings represent an alternative strategy for the treatment of DMD. The mechanism of myosin inhibition is structural in nature and, as such, is independent of inflammation or the specific genetic lesions conferring disease. As a result, we predict that it should be complimentary with therapeutic approaches that alter inflammation or modify/replace the dystrophin gene, such as antisense oligonucleotides or AAV microdystrophin. Further studies will explore whether EDG-5506 confers additional therapeutic benefit when combined with glucocorticoids or genetic strategies. Importantly, short-term clinical studies have now been completed in healthy volunteers and individuals with Becker muscular dystrophy using EDG-5506 (NCT04585464), and longer-term studies are underway in Becker muscular dystrophy and DMD (NCT05291091 and NCT05540860).

## Methods

### Actin and myosin S1 preparation.

Smooth muscle chicken gizzard S1 was purchased from Cytoskeleton Inc. Other myosin S1 was prepared via published protocols (21). Briefly, fresh rabbit psoas and frozen porcine ventricular muscle (Pel-freez) were extracted in 0.3 M KCl, 0.15 M KH_2_PO_4_, 1 mM EGTA, 1 mM ATP, 1 mM DTT, pH 6.5, at 4°C. Full-length myosin was purified from each extract via low-ionic precipitations with final precipitation into PM12 buffer (12 mM PIPES, pH 7.0, 2 mM MgCl_2_). Purified myosin was resuspended in PIPES/EDTA buffer (20 mM PIPES, pH 6.8, 10 mM EDTA, 1 mM DTT) and reacted with 0.2 mg/mL α-chymotrypsin for 30 minutes at room temperature. Proteolysis was quenched with PMSF (final concentration 100 μM), and the reaction was centrifuged for 45 minutes at 230,000*g*. S1 fragment in the supernatant was dialyzed into PM12 with 0.02% sodium azide and clarified by centrifugation. Sucrose was added to 10% w/v and flash-frozen in liquid N_2_ and stored at –80°C. Actin was purified from minced porcine cardiac muscle after 10-minute incubation in extraction buffer (0.5 M KCl, 0.1 M K_2_HPO_4_, pH 7.0). Centrifuged pellets were washed 3–4 times with carbonate buffer (Na_2_CO_3_, pH 8.2–8.5) and then 3 times with cold acetone. Residues were collected and dried to yield acetone powder. Actin was purified using published methods (21). Actin was polymerized with 0.1 volumes of 10× polymerization buffer (50 mM PIPES, pH 7.0, 550 mM KCl, 22 mM EGTA, 22 mM MgCl_2_, 10 mM ATP) and incubated at room temperature for 60 minutes.

### Myofibril preparation.

Myofibrils were prepared from various animals and tissues: rabbit psoas muscle and porcine cardiac muscle were purchased from Pel-Freez Biologicals. Human bicep and soleus muscle were purchased from BioIVT, and neonatal rat muscle was obtained in house. All myofibrils were prepared using published methods (21).

### Myofibril and myosin S1 enzymatic activity.

A lactate dehydrogenase/pyruvate kinase enzyme-coupled system (62) was used to monitor ATP hydrolysis rates in various muscle preparations. Rabbit psoas myofibrils were used for high-throughput screening at pCa 6.25 at 0.25 mg/mL. A multidrop cassette (Thermo Fisher Scientific) dispensed 10 μL of the coupled enzyme system, calcium, and myofibrils to 10 μL ATP, NADH and PEP to initiate the reaction prior to reading on a plate reader (Envision, Perkin Elmer) for 10 minutes per plate at room temperature. Hits were reconfirmed, and secondary assays were used to evaluate selectivity, including bovine cardiac myofibrils (pCa 5.86) and enzyme coupled hits with a hexokinase screen (Sigma-Aldrich).

### Stopped-flow analysis of Pi release from myosin.

The rate of Pi release from myosin with actin utilized a fluorescent phosphate binding protein, as described previously (63). Experiments were performed in 10 mM MOPS, pH 7.0; 5 mM KCL; 1 mM EGTA; 1 mM DTT; 0.5 mM MgCl_2_. ATP and ADP were removed by incubation with apyrase (64). Prior to mixing with 1 mM ATP in a stopped flow device, compound was incubated with 6 μM psoas S1 at 25°C for 30 minutes.

### Animal models.

C57BL/6 (stock no. 000664), C57BL/10J (stock no. 000665, control for *mdx* experiments), C57BL/10ScSn-Dmd^mdx^/J (*mdx*, stock no. 001801), and D2.B10 (DBA/2-congenic) Dmd^mdx^ (DBA/2J *mdx*, stock no. 013141) mice were from The Jackson Laboratory. Sprague-Dawley rats were purchased from Charles River Laboratories. For ex vivo experiments, mice were 10–16 weeks old. For in situ experiments, mice were 8–14 weeks of age. For in vivo chronic dosing studies, *mdx* and DBA/2J *mdx* were 4–5 weeks of age at study start. Dosing was via a 10 mL/kg suspension (1% methyl cellulose, 0.1% Tween 80 in water). For studies in 14-day-old preweaning mice, PO dosing was via syrup suspension (3 mg/kg/d). GRMD were supplied by Texas A&M Department of Veterinary Integrative Biosciences.

### Skinned fiber force measurements.

Measurement of isometric force in skinned fibers from rabbit psoas, rat soleus, and rat cardiac trabeculae were performed as described previously (65) with an 802D fiber system (Aurora Scientific). Muscle bundles were thawed from –80°C and placed in relaxing solution. Single fibers were transferred to the testing apparatus with steel pins connected to a force transducer and motor (model 403A and 322C, Aurora Scientific), and collodion glue was applied. Temperature was set to 15°C for skeletal fibers and 30°C for rat cardiac trabeculae. The wells of the 802D were filled with different ratios of relaxing and activating pCa solutions as detailed previously (65). All control solutions contained 1% final DMSO. Traces were compared between control and compound runs to evaluate isometric force and calcium sensitivity.

### Ex vivo force measurement.

Isometric force was measured ex vivo in mouse EDL and soleus muscle. EDL muscle eccentric injury was based on published protocols (30), with degree of lengthening and stimulation modified to yield a uniform drop in *mdx* isometric and peak force over 10 contractions. After isolation, muscle was mounted via 5-0 suture to a combination servomotor/force transducer (300C-LR, Aurora) between 2 electrodes connected to a stimulator (701C, Aurora) in Ringers solution at 27°C with continuously infused CO_2_/O_2_ gas mixture. Experiments were recorded with custom MATLAB software via a National Instruments PCIe-6321 board and 2090A BNC connector. Muscles were preincubated for 60 minutes with DMSO (final 0.1%)/compound before 10 eccentric contractions, 1 minute apart, with 100 milliseconds of isometric stimulation at 100 Hz and active lengthening of 0.1 L_0_ at a rate of 2 L_0_/s. After lengthening, muscles were held at 1.1 L_0_ with stimulation for 100 milliseconds (Supplemental Figure 2A).

For procion imaging, injury protocols were performed in 1% procion MX Dye (020 Brilliant Orange, Jacquard Products) and fixed in cold 4% paraformaldehyde and then cold 30% sucrose/PBS before cryosection. Laminin immunohistochemistry used rabbit polyclonal (L9393, Sigma-Aldrich, 1:20) and chicken anti-rabbit Alexa Fluor 488 (Invitrogen, 1:500). Slides were scanned at ×20 with Nikon Te-2000 dark-field microscopy and analyzed with Imaris software (Bitplane AG). Imaris’ surface component algorithm was used to detect procion-labeled fiber boundaries. Nonspecific procion staining of the peripheral tendon and outer layer of cells (where handling could cause injury) was excluded by applying a size and location filter (>2× average fiber size and located adjacent to laminin-positive fibers at the edge of the muscle). Each fiber was identified with an ID and color coded before calculation.

### Ex vivo analysis of mdx mouse lumbrical muscle contraction.

Intracellular calcium quantification and isometric injury were described previously (31). Lumbrical muscles were transferred to a custom-built chamber and mounted horizontally to a force transducer (model 400A, Aurora Scientific) at 25°C. Sarcomere length was adjusted to 2.5 μm by microscope. Contraction was elicited with 0.2-millisecond stimuli and injury via 1-second stimuli at 125 Hz.

For fluorescence experiments, a 75W xenon lamp was used with wavelengths selected using a diffraction grating monochromator (model DeltaRAM, Photon Technology International). Muscles were loaded with mag-fura-2 AM (10 μM in Tyrode) for 30 minutes at 25°C, excited at 344 and 375 nm (bandwidth 10 nm), and passed through a 510 nm emission filter (bandwidth 40 nm). Fluorescence responses were recorded alternately during 32 twitch contractions at 20-second intervals; background fluorescence was subtracted, and the response was averaged. High-affinity fura-2 was used to detect membrane breaches between tetanic contractions. Muscles were incubated with fura-2 AM (15 μM in Tyrode) for 30 minutes before 12 injurious contractions, separated by 1 minute with (10/s) alternating excitation wavelengths of 340 and 375 nm (bandwidth 1.25 nm) with a 510 nm emission filter (bandwidth 40 nm) and preloading background subtraction. Still and video images were captured using a digital camera (Nikon D750).

### In situ force measurement.

Measurement of isometric force and eccentric injury response in situ was performed in mouse TA muscle based on published protocols (30). Mice were anesthetized with 2% isoflurane (0.7%–1.5% maintenance) on a 37°C platform. The distal tendon to the TA and EDL muscle was attached with 4-0 suture a servomotor (Aurora Scientific). The sciatic nerve was exposed at the knee for hook electrodes. For compound assessment, baseline isometric contraction was assessed at 25–175 Hz (9 mA, 300 ms) followed by 100 Hz stimuli every 5 minutes. After 15 minutes, compound was dosed PO, and recording continued for 4 hours followed by a second force frequency. For in situ eccentric injury, compound was administered PO, muscle was stimulated every 5 minutes at 100 Hz for 3.5 hours, and then 2 lengthening contractions were performed (10 min apart, 100 ms at 150 Hz, then lengthening 0.2 L_0_ at 2 L_0_/s and hold for 100 ms). Isometric force before and 10 minutes after the injury contractions was recorded.

### Phosphoprotein array analysis.

*mdx* mouse EDL muscle was prepared for ex vivo analysis as described (*n* = 5). Muscles were incubated for 1 hour with DMSO or EDG-5506 (5 μM) and then flash frozen in liquid nitrogen. Tissue preparation and analysis were performed as recommended by the manufacturer (Cell Signaling Phospho Antibody Array, Full Moon Biosystems).

### In vivo assessments in mice.

Prior to in vivo assessments, mice were dosed PO after a 2-hour fast with 10 mL/Kg suspensions. Grip strength and rotarod assessments were performed masked, 4 hours after dosing using published protocols (66, 67). Grip strength was performed with a Columbus Instruments Rodent Force Meter. Forepaw strength represented the average of 5 trials with 30-second rest periods, normalized by BW. Rotarod endurance was performed with a Columbus Instruments RotaMax. Mice were placed on the device drum at 1 rpm, which accelerated 1 rpm every 3 seconds to 12 rpm and a maximal time of 500 seconds. This was repeated 3 times with a 3-minute rest and total time reported. Voluntary wheel running performance was assessed with a customized system. Timestamped RPMs were measured 24 hours per day by infrared sensors, and a custom software calculated distance. Whole-body EBD was visualized after intravenous administration (35). EBD in PBS at 10 mg/mL was sterile filtered, and 50 μL/10 g BW was injected via tail vein. After 24 hours, animals were sacrificed, skin was removed and fixed in 4% formalin for 24 hours, and photographed using a Leica/Lumix camera.

### Plasma CK activity.

Plasma from mice and dogs were analyzed for CK activity using standard protocols (Pointe Scientific, Thermo Fisher Scientific). Note that experiments performed in Supplemental Figure 2D and Supplemental Figure 4B were performed with separated batches of *mdx* and CK kits and exhibited higher background CK activity compared with previous experiments (Figure 2H and Figure 4B).

### Histological and immunohistochemical analysis.

For sterile muscle injury, mice were anesthetized with isoflurane and 50 μL cardiotoxin (12 × 10^–6^ M, 217503, Millipore) injected in the TA muscle. Muscles 8 days after injury were snap frozen in nitrogen-chilled isopentane. 8 μm cryosections were cut and stained with H&E. For each analysis, more than 10 slides (per condition/group) containing 6 muscle sections/sample were used, and myofibers in the injured area were counted and measured with a Mirax digital scanner and HALO software (68). For adult mouse studies, muscles were fixed in 10% formalin and embedded in paraffin for sectioning. Laminin staining (RB-082, Thermo Fisher Scientific) was used to outline fibers, and picrosirius red was used for collagen accumulation. Image analysis was performed using ImarisX64 software (Bitplane AG), and fiber sizing was calculated using minimal Feret’s diameter (69). For after weaning juvenile mouse studies, cryosections from soleus muscles were mounted in ProLong antifade mountant with DAPI (Thermo Fisher Scientific). Fiber type, size, and central nucleation were quantified with analysis via SMASH (70) and ImageJ (NIH). Antibodies used were mouse anti-MHC2A (1:50, SC-71, Developmental Studies Hybridoma Bank) and mouse anti-eMHC (1:10, BF-45, Developmental Studies Hybridoma Bank).

### RNA-Seq analysis of gastrocnemius muscle.

RNA-Seq was performed with isolated single nuclei using the 10X Genomics Chromium Single Cell 3’ Protocol, Accessories, and Kits (CG000183 Rev A). Data were then analyzed by Rosalind (https://rosalind.onramp.bio/), with a HyperScale architecture developed by OnRamp BioInformatics Inc. Data have been submitted to the Gene Expression Omnibus (GEO) database at the NIH (accession GSE227510).

### Myosin isoform analysis.

Biopsies of dog gastrocnemius muscle (GRMD, *n* = 5; age, 6–9 months; WT, *n* = 5; age 6–7 months), rabbit psoas, and bovine masseter were snap frozen on liquid nitrogen. Muscle was thawed in lysis buffer (30 mM Tris, pH 7.5, 1% SDS) and homogenized in a bead beater for 3 minutes at 58 cycles per second. Myosin-separating gels were prepared and run as described previously (71) using 1 μg protein lysate for gastrocnemius and 3.5 μg psoas/masseter (at 80 V for 8 hours and 85 V for 18 hours) at 4°C. Protein was quantified using Sypro Total Protein stain (rapid protocol) and visualized on a LiCor Odyssey M. Quantification of fast and slow MHC was performed using Empiria Studio 2.2 software (LiCor Inc.).

### Habitual activity and circulating biomarker measurement in GRMD.

Studies were performed in two phases. In the first phase, GRMD (*n* = 4, female, 7 months old) were dosed PO daily with vehicle for 2 weeks, followed by a suspension of EDG-5506 (3 mg/kg daily for 2 days, then 1 mg/kg daily for 2 weeks) and then with vehicle for 2 weeks. Blood was drawn at regular intervals (2–3 draws during the vehicle baseline, 5–8 draws during the dosing period, and 2 draws during the vehicle washout). In a separate dosing period, the same 4 dogs (10 months old) were administered vehicle for 14 days with regular blood draws (6–9 for each dog across the 14-day period). CK activity (Pointe Scientific, Thermo Fisher Scientific) was measured from plasma. In the second phase, the same 4 dogs (15 months old) were fitted with a collar-bound activity monitor (Fitbark 2, Fitbark Inc.) as described previously (42), and baseline activity was recorded for 27 days before daily oral gavage with vehicle for 22 days. Three dogs were then dosed in a similar pattern to phase 1 with EDG-5506 (2 mg/kg for 4 days and then 2 mg/kg every other day for 7 days). The fourth dog was dosed with vehicle over the same period. Dogs were then returned to vehicle for a further 12 days. Average daily activity (“Fitbark points”) and time performing various activities (play, rest, active) were recorded.

### Somascan plasma proteomics analysis.

Somascan (SomaLogic) protein array (1,305 protein analytes for human plasma) was used with dog plasma. Samples were processed according to SomaLogic’s standard protocols (Plasma_4.3_20180208_1.5k) as described previously (72). Raw Somascan data were hybridization-control normalized and median-signal normalized and sorted into populations of WT, GRMD baseline, EDG-5506 treatment, and postdosing washout. Two WT samples were taken from one age-matched healthy littermate. GRMD baseline and EDG-5506 treatment samples were taken from 2 of 4 dogs, with 2 GRMD baseline samples taken per dog the week prior to starting EDG-5506 and treatment samples from 3–4 samples per dog taken across the treatment period. Two postdosing washout samples were taken per dog in the week following treatment. Population RFU values were log-transformed to reduce heteroscedasticity and then averaged for every target. Relative concentrations for each target were calculated by subtracting the log-transformed data from that of the WT population. Target comparisons were made using a using a 2-tailed *t* test, correcting for the false discovery rate using the Benjamini-Hochberg method (73).

### Statistics.

Unless indicated, data are shown as the mean ± SEM, and statistical significance was calculated using 1-way ANOVA, 2-tailed Student’s *t* test, or the Kolmogorov-Smirnov test. Where appropriate, *P* values have been corrected for multiple comparisons. *P* values of less than 0.05 were considered significant.

### Study approval.

Mouse procedures at University of Colorado, Boulder, were performed in accordance with and with approval of the IACUC (protocol 2589). Sterile injury experiments were carried out in accordance with and with approval of the IACUC at Johns Hopkins University (license MO18C251). All dogs were used and cared for according to principles outlined in the *Guide for the Care and Use of Laboratory Animals* (National Academies Press, 2011). Procedures were approved by the Texas A&M IACUC (protocols 2018–0182 and 2018–0393).

## Author contributions

AJR, KK, SVB, PN, HLS, LAL, SS, KH, and LN conceptualized the study. HMR, CV, DRC, BR, and KK provided methodology. HMR, MD, BB, YQ, AKP, BLNS, KH, SS, BR, AVD, CV, YS, DRC, AR, AP, MM, TIJ, JY, and YEL provided investigation. LN, HLS, AJR, LAL, ERB, and KK supervised the study. AJR, BB, and AKP wrote the original draft of the manuscript. BB, MD, LAL, LN, and HLS reviewed and edited the manuscript.

## Supplementary Material

Supplemental data

Supplemental data set 1

Supplemental data set 2

Supplemental data set 3

Supplemental data set 4

Supplemental data set 5

Supplemental data set 6

Supplemental video 1

Supplemental video 2

Supplemental video 3

## Version 1 03/30/2023

In-Press Preview

## Version 2 05/15/2023

Electronic publication
