# Supplementary material for: Modulating fast skeletal muscle contraction protects skeletal muscle in animal models of Duchenne muscular dystrophy
Source: J Clin Invest. 2023 May 15;133(10):e153837. doi: 10.1172/JCI153837 (PMC10178848; doi:10.1172/JCI153837)
Supplement: Supplemental data [file jci-133-153837-s034.pdf]

Modulating fast skeletal muscle contraction protects skeletal muscle in animal models of Duchenne muscular dystrophy.

Supplemental Section

Supplemental Table 1. Efficacy of EDG-5506 against myofibril preparations from additional muscle sources.

ATPase Source	Myosin Composition	IC_{50} (μM)	Maximum Inhibition (%)[a]
Myofibrils			
**Rabbit Cardiac**	25% MHCα, 75% MHC β(1) (MYH6, MYH7)	> 100	--
**Neonatal Rat (P1)**	100% neonatal/embryonic MHC (MYH3, MYH8)(2)	0.2	96
**Human Bicep**	50% Type I (MYH7), 50% IIa, IIx (MYH2, MYH1)	1.2	89
**Human Soleus**	66% Type I (MYH7), 33% IIa, IIx (MYH2, MYH1)	1.1	79

[a] "--" indicates an undefined value

Myofibril assays were all performed at the pCa 50 specific to each muscle and S1 assays were run with 9.5 μM F-actin. The reported IC_{50} value is the point of 50% inhibition relative to the vehicle control, which was assigned a normalized value of 100%. The maximum inhibition, where reported, is the bottom plateau of the 4-parameter logistic fit.

1. Marian, A.J. On mice, rabbits, and human heart failure. *Circulation* **111,** 2276-2279 (2005)
2. D'Albis, A., Couteaux, R., Janmot, C. & Roulet, A. Specific programs of myosin expression in the postnatal development of rat muscles. *Eur J Biochem* **183,** 583-590 (1989)
3. Setsuya, M., Minobi, W., Bristow, M.R., Leinwand, L.A. Myosin Heavy Chain Isoform Expression in the Failing and Nonfailing Human Heart. *Circ Res* **86**, 386-390 (2000)

Supplemental Table 2. Efficacy of EDG-4131 against myofibril preparations from additional muscle sources.

ATPase Source	Myosin Composition	IC_{50} (μM)	Maximum Inhibition (%)[a]
Myofibrils			
**Rabbit Psoas**	96% Type IIx (MYH1) (1)	1.8	98
**Rabbit Psoas (S1 Fragment)**	96% Type IIx (MYH1)	0.7	> 95
**Bovine Masseter**	100% Type I (MYH7) (2)	>100	--
**Mouse Gastroc**	21% IIa (MYH2), 15% IIx (MYH1), 56% IIb (MYH4) (3)	0.6	98
**Mouse TA**	Variable reported levels of IIa (MYH2), IIx (MYH1) and MYH4 (3, 4)	0.7	> 99
**Porcine Ventricle**	100% MHC β (MYH7) (5)	> 100	--
**Rabbit Cardiac**	25% MHCα, 75% MHC β(1) (MYH6, MYH7)	> 100	--
**Human Cardiac**	7% MHCα, 93% MHCβ[3]	> 100	--
**Neonatal Rat (P1)**	100% neonatal/embryonic MHC (MYH3, MYH8)(2)	0.8	98
**Human Bicep**	50% Type I (MYH7), 50% IIa, IIx (MYH2, MYH1)	1.0	91
**Human Soleus**	66% Type I (MYH7), 33% IIa, IIx (MYH2, MYH1)	2.4	80

Supplemental Table 3. Selected Pathways Represented by Proteins which were Elevated at Baseline and Decreased with EDG-5506 Treatment via Somascan analysis.

Pathway Name	Reactome Reference	Entities (Found/Total)	FDR-Corrected P-Value
**Apoptosis**	**R-HSA-109581**	**6/182**	**< 0.05**
Activation of BH3-only proteins	*R-HSA-114452*	*4/30*	*< 0.01*
**Immune System**	**R-HSA-168256**	**30/2265**	**< 0.01**
Cytokine Signaling in Immune System	*R-HSA-1280215*	*16/821*	*< 0.01*
Innate Immune System	*R-HSA-168249*	*12/1201*	*< 0.01*
**Metabolism**	**R-HSA-1430728**	**21/2143**	**0.051**
Gluconeogenesis	*R-HSA-70263*	*3/34*	*0.013*
Glycolysis	*R-HSA-70171*	*4/78*	*0.014*
Metabolism of nucleotides	*R-HSA-15869*	*4/103*	*0.028*
**Gene Expression (Transcription)**	**R-HSA-74160**	**17/1542**	**0.124**
TP53 Regulates Metabolic Genes	*R-HSA-5628897*	*6/88*	*< 0.01*
**Cellular Responses to External Stimuli**	**H-HAS-8953897**	**14/783**	**0.011**
Detoxification of Reactive Oxygen Species	*R-HSA-3299685*	*2/39*	*0.09*
Cellular Response to Heat Stress	*R-HSA-3371556*	*6/95*	*< 0.01*
**Muscle Contraction**	**R-HSA-397014**	**4/204**	**0.124**
Ion Homeostasis	R-HSA-5578775	3/54	0.03
**Signal Transduction**	**R-HSA-162582**	**27/2599**	**0.061**
Akt Phosphorylates Targets in the Cytosol	*R-HSA-198323*	*2/14*	*0.023*
mTOR Signaling	*R-HSA-165159*	*4/41*	*< 0.01*

Proteins which were decreased in response to EDG-5506 treatment were analyzed for pathway representation using the Reactome database. Top-level pathway families are shown in bold, while individual pathways within those groups are shown in italics. P-values were calculated using a binomial test and corrected for the false-discovery rate using the method by Benjamini and Hochberg.

Supplemental Figure 1 (Part 1)

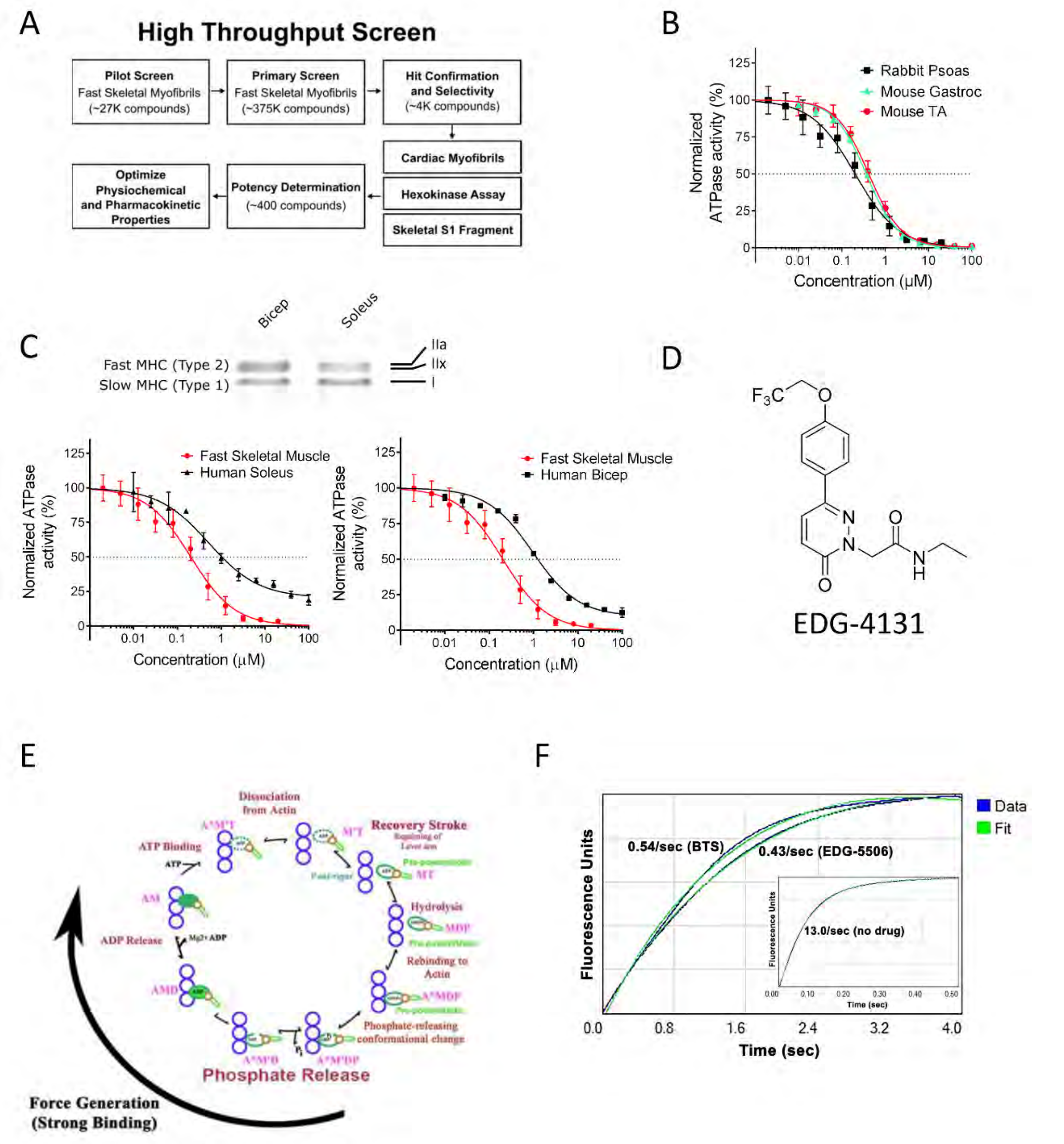

EDG-5506 is a selective inhibitor of fast skeletal myosin ATPase and force generation in fast skeletal muscle. (A) Illustrated workflow employed for the identification of selective inhibitors of fast skeletal muscle myosin. Confirmed hits from the pilot and primary screen using rabbit psoas myofibrils were advanced into selectivity assays using cardiac ventricle myofibrils, and specificity assays using myosin S1 fragments from rabbit psoas. Selective compounds, ranked by potency, were then optimized for drug-like properties. (B) Activity and enzymatic activity of EDG-5506 in purified myofibrils with different fast skeletal myosin composition. Errors +/- SD. (C) Top, Human muscle myosin composition measured by electrophoresis. Bottom, ATPase activity curves for EDG-

5506 with myofibrils isolated from human bicep (left) and soleus (right) muscle. Inhibition curve for rabbit psoas muscle included for comparison. Errors +/- SD. Note that the ATPase of fast muscle is approximately four times that of slow (data not shown), accounting for the greater than proportional inhibition of these mixed fiber preparations with EDG-5506. (D) Chemical structure of EDG-4131. (E) The actin-myosin ATPase cycle depicting the point in the cycle that actin binding triggers the release of inorganic phosphate (Pi). Force generation is thought to begin at this point, when myosin that is bound weakly to actin undergoes a conformational change to allow stronger association with actin. This creates a route for inorganic phosphate to escape from myosin, where it is trapped following ATP hydrolysis (3). (F) EDG-5506 and N-Benzyl-p-toluenesulfonamide (BTS) (both 10 μM) inhibit Pi release from fast skeletal muscle myosin. Stopped flow analysis of Pi release from skeletal myosin S1 measured by increased fluorescence accompanying binding to a labeled phosphate binding protein (4).

Supplemental Figure 1 (Part 2)

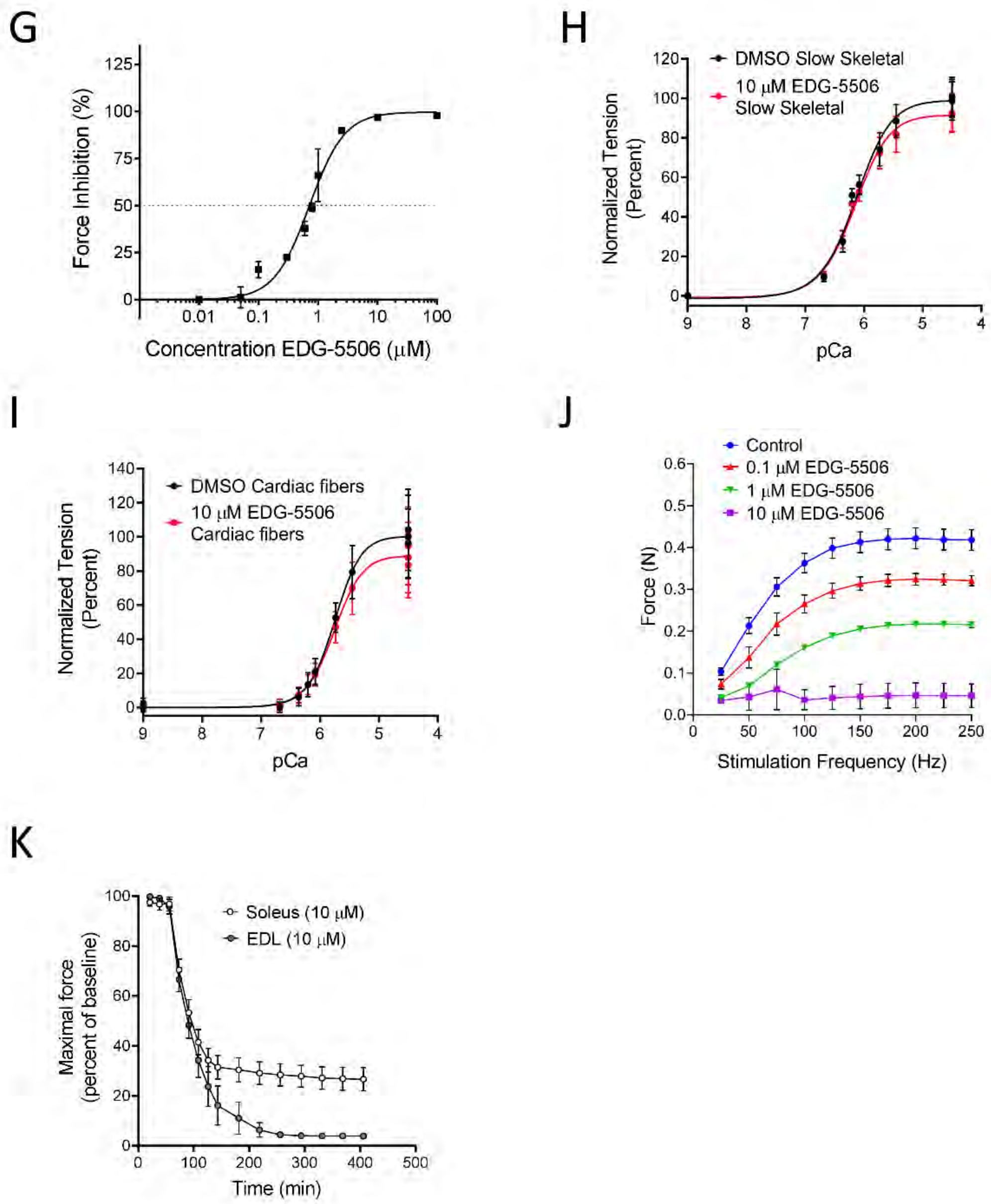

EDG-5506 is a selective inhibitor of fast skeletal myosin ATPase and force generation in fast skeletal muscle. (G) Effect of different concentrations of EDG-5506 on maximal force (at pCa 4.5) in single skinned rabbit psoas fibers. All concentrations are normalized to DMSO control (each point, N=4-6), Errors +/- SEM. (H) Force-calcium curve in single permeabilized slow skeletal muscle fibers (rat soleus) with 10 μM EDG-5506 (N=5). (I) Force-calcium curve in single permeabilized cardiac muscle trabecular strips (rat) with 10 μM EDG-5506 (N=5). Errors +/-SEM. Recorded decrease in maximal force is non-significant. (J) Force/frequency relationship in mouse EDL muscle *ex vivo* after treatment with different concentrations of EDG-5506 (N=3-5). Errors +/-SEM. (K) Percent of initial force with time after addition of EDG-5506 in WT mouse EDL vs soleus muscle *ex vivo* (EDL, N=2, SOL N=3. Mouse SOL is approx. 37% slow, 63% fast (5)). Force was recorded at 250 Hz every 18-38 mins. Each point represents mean peak force +/- SEM.

Supplemental Figure 2

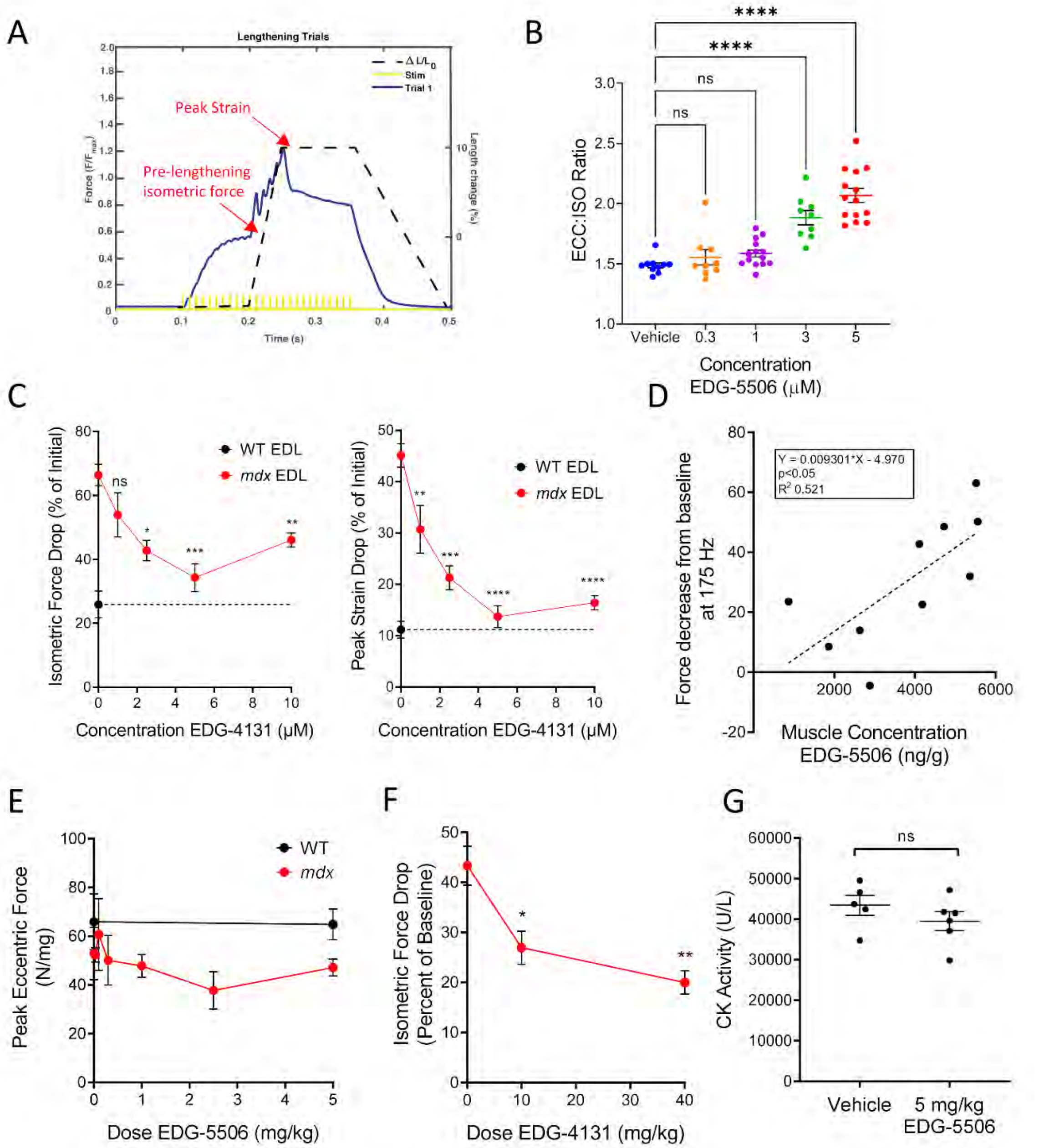

Strength loss during eccentric contraction of dystrophic muscle is dependent upon contraction via myosin. (A) Graphic illustrating the eccentric injury protocol in mouse EDL muscle *ex vivo*. Key readouts are indicated by the red arrows. (B) Ratio of eccentric peak strain to isometric force in *mdx* EDL muscle *ex vivo* one-hour after incubation with vehicle or the indicated concentrations of EDG-5506 (N=9-14). (C) Isometric force (left) and peak strain (right) drop from the first to the last contraction in *mdx* EDL muscle *ex vivo* as a function of EDG-4131 concentration (N=3-15 per point). (D) Relationship between EDG-5506 muscle concentration (ng

EDG-5506 per g TA muscle) and *in situ* force decrease in WT mice 2.5 hrs post oral administration. (E) Peak eccentric force three-hours after oral administration of vehicle or the indicated dose of EDG-5506. (F) Effect of oral doses of EDG-4131 on isometric force drop 10 min after two lengthening contractions of TA muscle in *mdx* mice *in situ*, represented as a percent of pre-injury force. (G) CK activity four-hours post oral administration of vehicle or EDG-5506 in the absence of eccentric contractions (*in situ* sham) (N=5-6). Graphs show mean +/- SEM. Significance calculated by one-way ANOVA with Tukey's multiple comparison correction. ****$p<0.0001$

Supplemental Figure 3

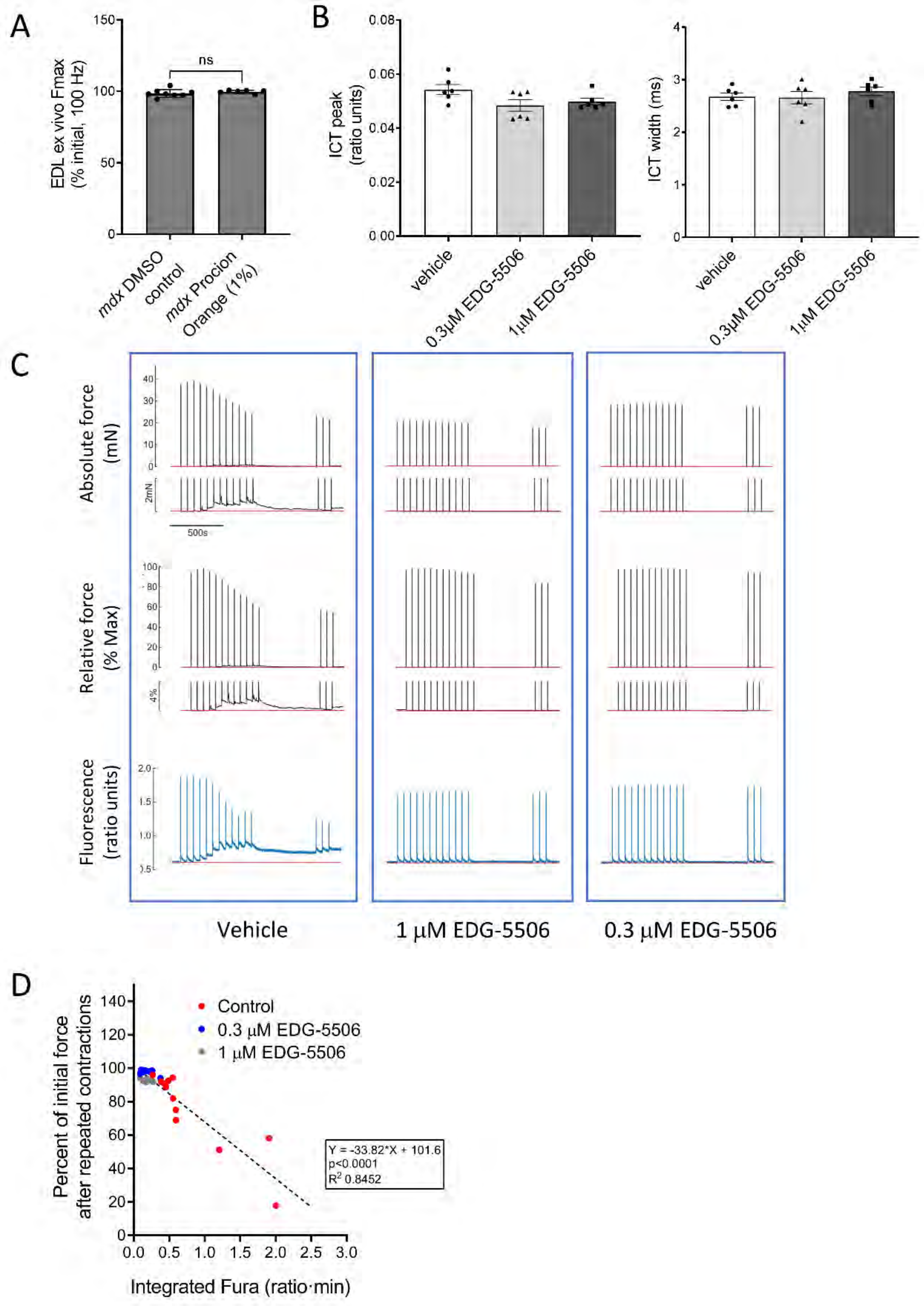

Membrane injury arising from contraction of dystrophic muscle is dependent upon contraction via myosin. (A) Percent of initial force 60 min after addition of vehicle or 1% procion orange in *mdx* mouse EDL muscle *ex vivo*. Force was recorded at 100 Hz. Each point represents mean peak force (N=6-8). (B) Left, intracellular calcium transient (ICT) peak during twitch contractions and right, ICT width (full width at half maximum) measured by mag-fura-2 fluorescence response after 1 hr incubation with DMSO or EDG-5506 in WT mouse lumbrical muscle (N=6). (C) Representative force (black) and fura-2 fluorescence ratio (blue, measures intracellular Ca^{2+}) during 12 tetanic isometric contractions. Note the axis focus under the main force traces show increases in resting force only in vehicle treated muscle. (D) Correlation between percent force drop and average inter-contraction fura-2 fluorescence ratio during 12 tetanic contractions. Error bars shown +/- SEM. Significance calculated by one-way ANOVA with Tukey's multiple comparison correction.

Supplemental Figure 4

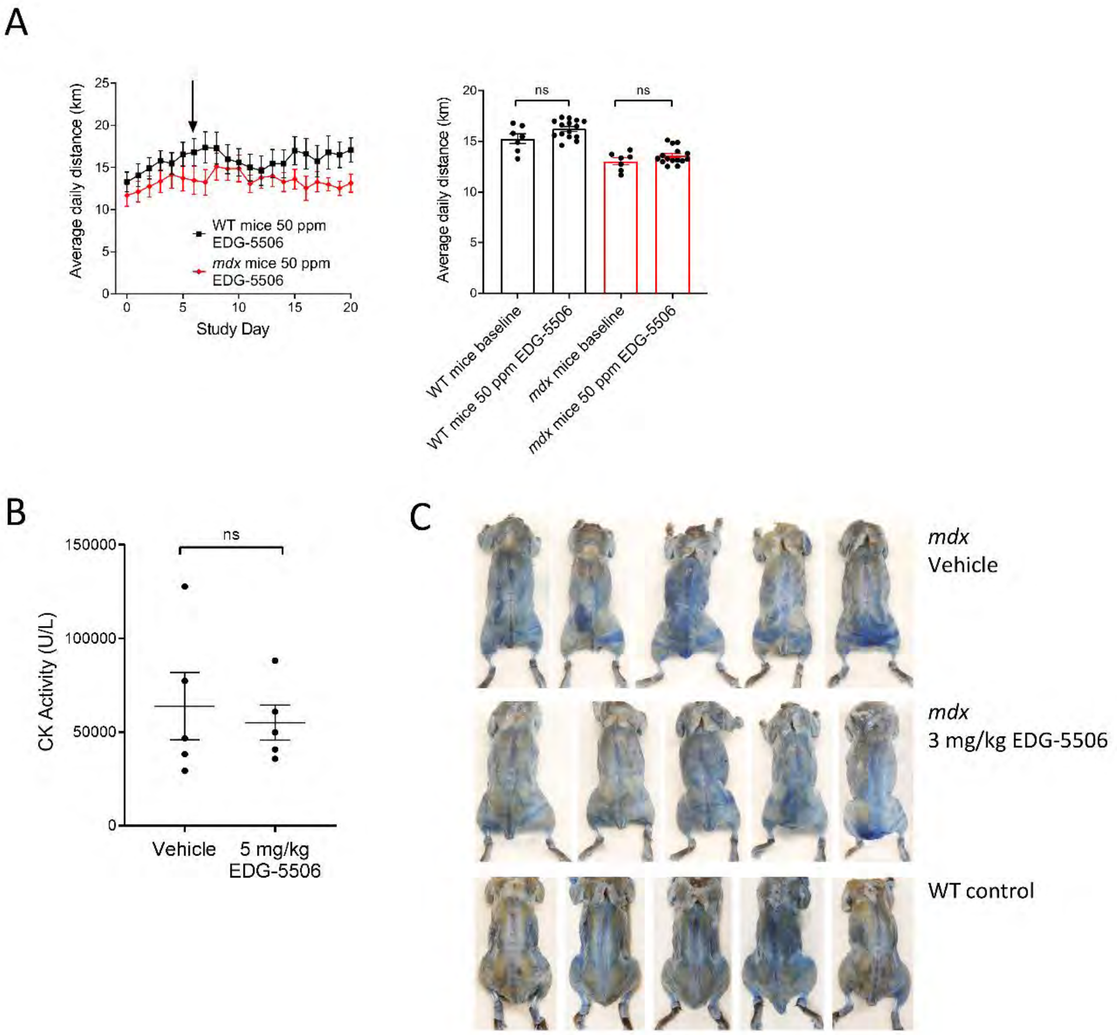

Normalization of membrane permeability with EDG-5506 in *mdx* mice without detrimental effects on strength and coordination in vivo. (*A*) Habitual wheel running in WT and *mdx* mice, Left, longitudinal data of average daily run distance. Arrow marks the switch from control chow to EDG-5506 chow (50 ppm or 0.13 mmol/Kg chow). Right, Average daily run distance pre- and post-administration of EDG-5506 50 ppm chow in WT and *mdx* mice, N=10. (*B*) Plasma CK activity in *mdx* mice 5 hours after a single dose of EDG-5506 in the absence of exercise (N=5). Note the higher baseline CK in this separate batch of *mdx* mice compared to Figure 4B). (C) Whole-body images of *mdx* and WT mice 24 hrs after intravenous administration of Evans blue dye. *Mdx* mice were treated daily for three weeks with vehicle or EDG-5506 via oral gavage. Errors +/- SEM. Significance calculated by one-way ANOVA with Tukey's multiple comparison correction.

Supplemental Figure 5 Part 1

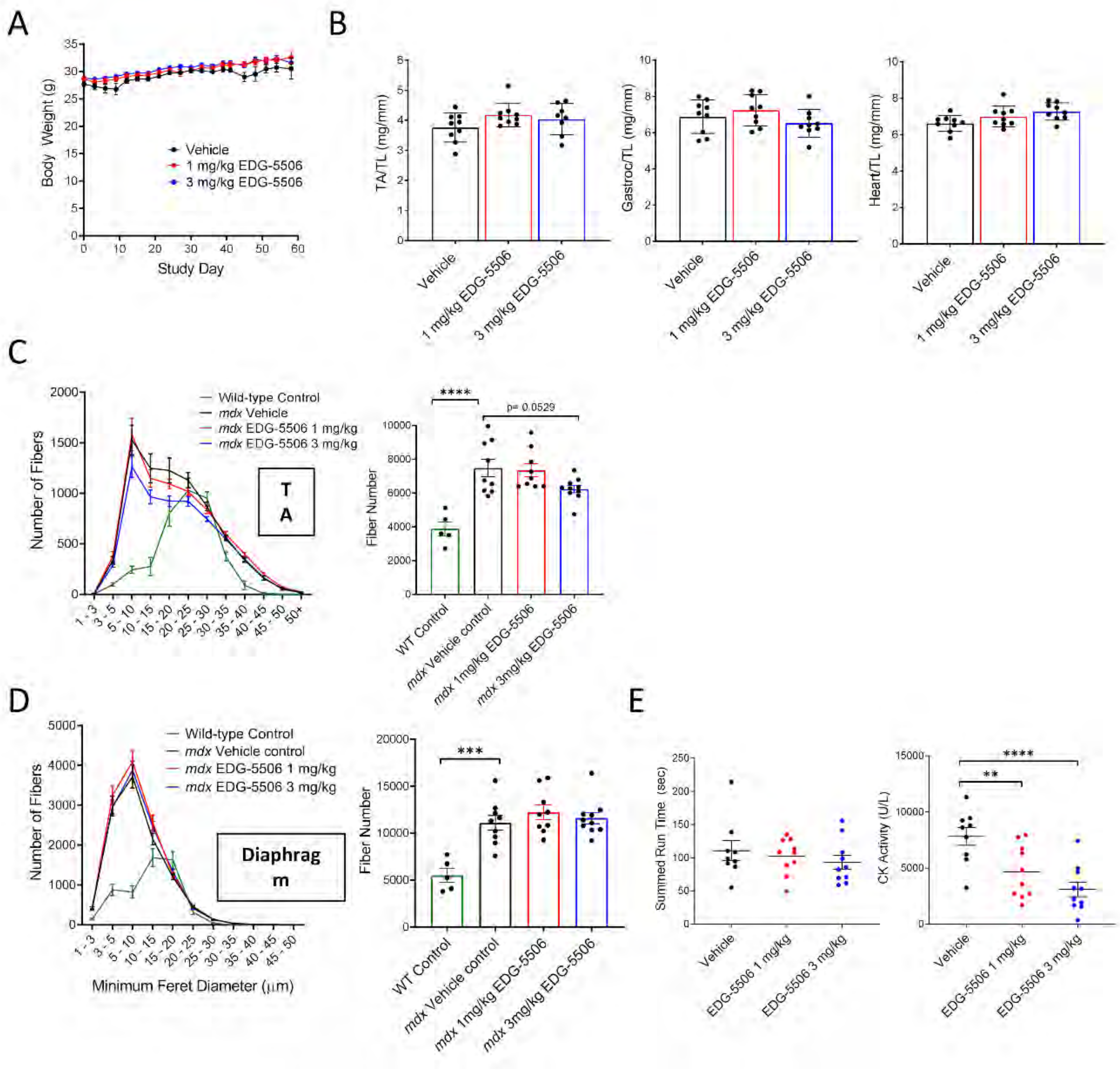

Longer term exposure of protective levels of myosin inhibition are sufficient to decrease muscle degeneration and fibrosis in *mdx* mice. (A-C) The effect of 8 weeks administration of EDG-5506 on body weight, muscle weight and skeletal muscle fiber size in *mdx* mice. (A) Average body weight during the eight-week dosing period in *mdx* mice. (B) Tibialis anterior (TA), gastrocnemius (Gastroc), and heart weight normalized by tibia length (TL) at the end of the eight-week study in *mdx* mice. (C) Minimum Feret's fiber diameter distribution of the TA muscle at the end of the eight-week study. (D) Minimum Feret's fiber diameter distribution of the diaphragm muscle at the end of the eight-week study. Shown is the fiber distribution and total number of fibers for each muscle analyzed (E) Rotarod run time (left) and plasma CK activity (right) from blood samples taken1 hour after the exercise intervention. Error bars shown +/- SEM. Significance calculated by one-way ANOVA with Dunnet's multiple comparison. *p<0.05; ** p<0.01; ***<0.001; **** p<0.0001. Each treatment, N=9-10

Supplemental Figure 5 Part 2

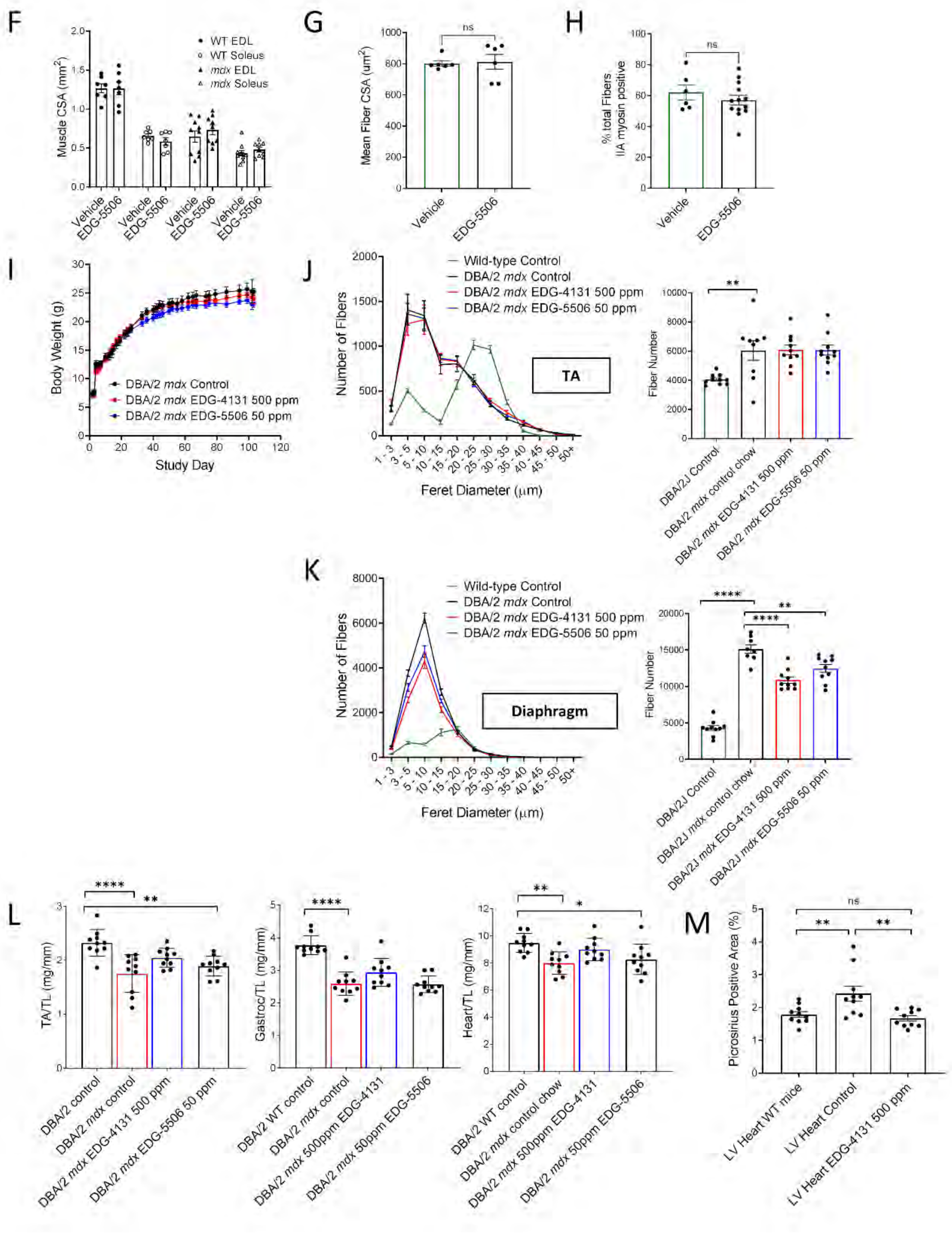

Longer term exposure of protective levels of myosin inhibition are sufficient to decrease muscle degeneration and fibrosis in *mdx* mice. (F-H) The effect of 3 weeks administration of EDG-5506 over the weaning period in *mdx* and WT mice. (F) Muscle cross sectional area of the EDL and soleus muscle. (G) Mean muscle fiber cross sectional area in *mdx* soleus muscle. (H)

Total proportion of IIa myosin positive fibers in *mdx* soleus muscle. (I-M) The effect of 12 weeks administration of EDG-5506 or EDG-4131 on body weight, muscle weight and skeletal muscle fiber size in DBA/2 *mdx* mice. (I) Average body weight during the dosing period. (J) Minimum Feret's fiber diameter distribution of the TA muscle at the end of the study. (K) Minimum Feret's fiber diameter distribution of the diaphragm muscle at the end of the study. Shown is the fiber distribution and total number of fibers for each muscle analyzed. (L) Tibialis anterior (TA), gastrocnemius (Gastroc), and heart weight normalized by tibia length (TL) at the end of the study. (M) Collagen quantification in the left ventricle in DBA/2 *mdx* mice after 12 weeks treatment with control or EDG-4131 chow (500 ppm). Error bars shown +/- SEM. Significance calculated by one-way ANOVA with Dunnet's multiple comparison. *p<0.05; ** p<0.01; ***<0.001; **** p<0.0001. Each treatment, N=9-10.

Supplemental Figure 6

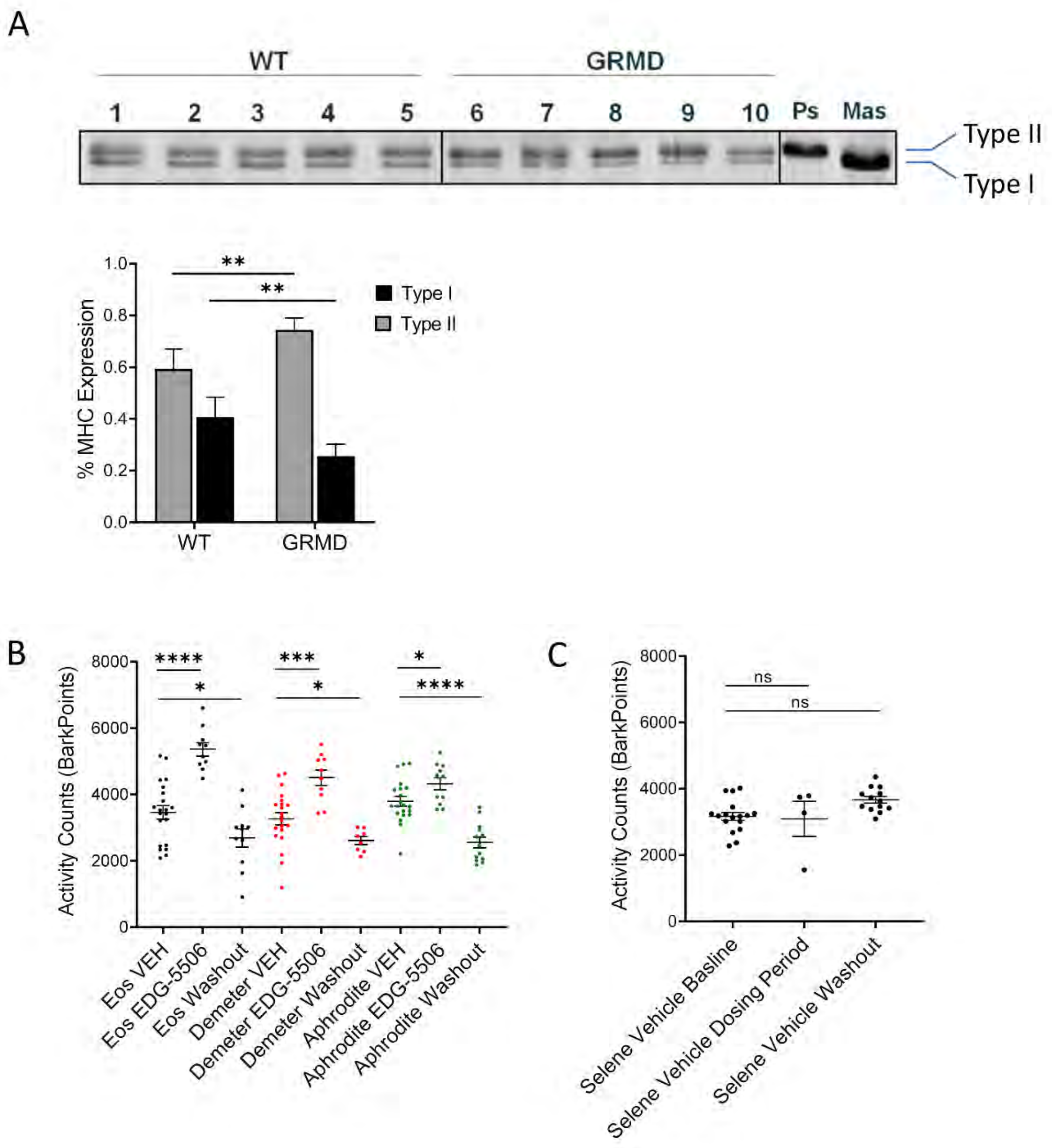

Selective inhibition of active contraction in fast skeletal muscle decreases CK and increases habitual activity in DMD dogs. (A) Upper panel, myosin heavy chain resolving gel of gastrocnemius homogenates derived from WT and GRMD dogs. Rabbit psoas (Ps) and bovine masseter (Mas) used as type II and type I loading controls. Lower panel, quantification of band density from the resolving gel. (B) Individual daily activity data using an electronic activity monitor (FitBark®) for the three DMD dogs dosed with EDG-5506 (15 mos old) before, during and after 11 days oral gavage with EDG-5506. Activity shown for 22 days dosing with vehicle (VEH). EDG-5506 dosing via capsule at 2 mg/kg daily for four days then every other day for an additional 7 days followed by vehicle for 12 days (Washout). (C) Individual activity data for a fourth dog dosed with vehicle during the same period. Significance calculated by unpaired t test. Error bars shown +/- SEM. *p<0.05; **p<0.01; *** p<0.001; **** p<0.0001.

Supplemental Figure 7.

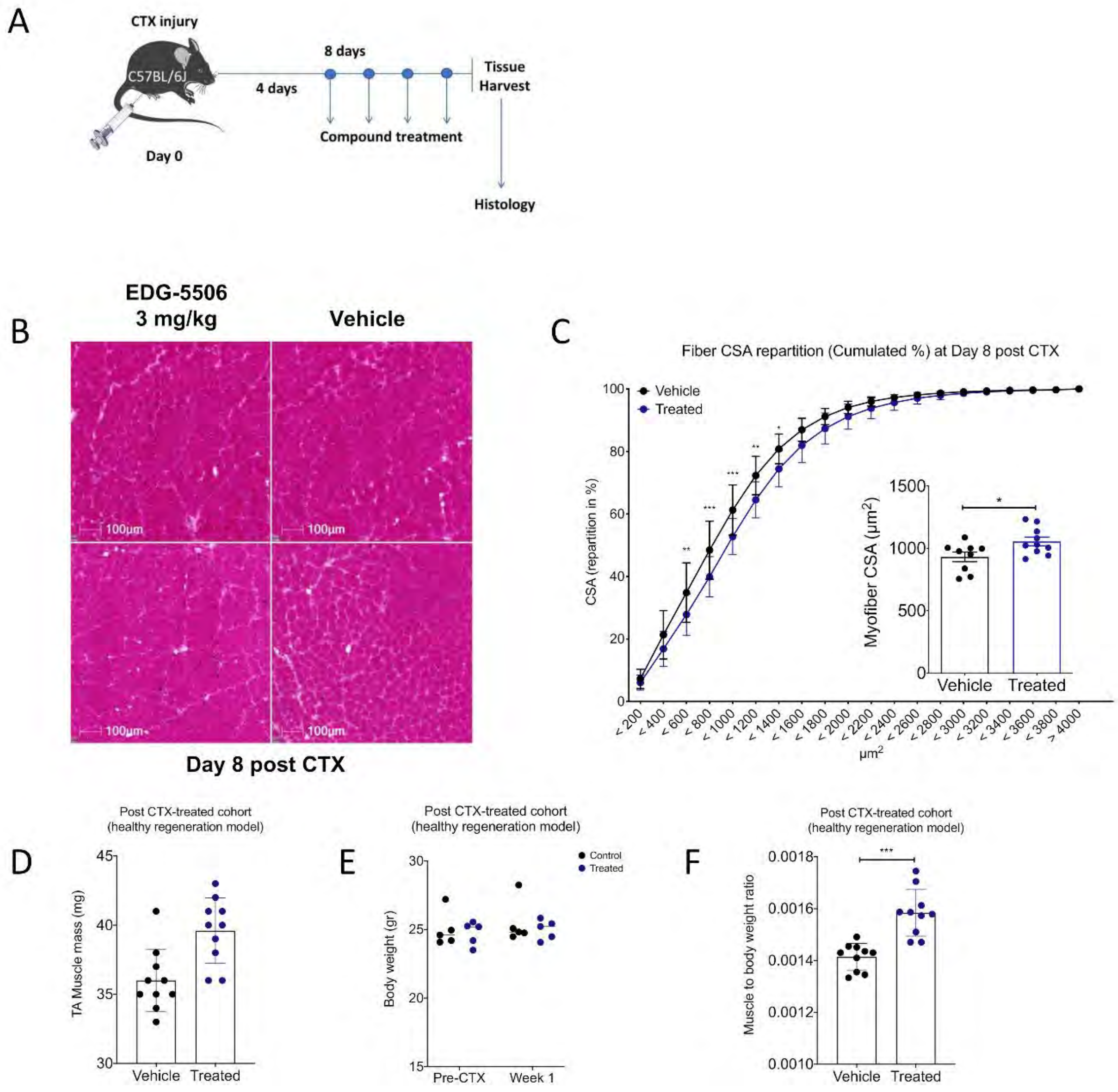

EDG-5506 treatment does not interfere with muscle repair after injury in healthy mice. (A) Study outline. Mice were administered an IM injection of cardiotoxin (CTX) into the TA muscle. EDG-5506 was administered daily starting from day 4 post-injury. At day 8 post-injury, muscles were removed for analysis. (B) Representative images of H&E stained TA muscle at day 8 post CTX-induced injury. (C) Fiber size repartition of regenerating muscle in vehicle-control, and EDG-5506 treated animals at day 8 post CTX injury. Insets show the average fiber CSA of regenerating muscle at day 8 post CTX injury (n=10 muscles per group). Significance calculated by two-way ANOVA. (D) TA muscle mass (in mg) at day 8 post CTX in vehicle-control, and EDG-5506 treated animals (n=10 muscles per group). (E) Body weight at day 8 post CTX in vehicle-control, and EDG-5506 treated animals (n=5 mice per group). (F) Normalized muscle to body weight ratio at day 8 post CTX in vehicle-control, and EDG-5506 treated animals (n=10). Significance calculated by two-

tailed Welch's t test. In all graphs, bars and lines represent mean ± SEM. *p<0.05; **p<0.01; *** p<0.001; **** p<0.0001.

1. Marian AJ. On mice, rabbits, and human heart failure. *Circulation.* 2005;111(18):2276-9.
2. d'Albis A, Couteaux R, Janmot C, and Roulet A. Specific programs of myosin expression in the postnatal development of rat muscles. *Eur J Biochem.* 1989;183(3):583-90.
3. Houdusse A, and Sweeney HL. How Myosin Generates Force on Actin Filaments. *Trends Biochem Sci.* 2016;41(12):989-97.
4. White HD, Belknap B, and Webb MR. Kinetics of nucleoside triphosphate cleavage and phosphate release steps by associated rabbit skeletal actomyosin, measured using a novel fluorescent probe for phosphate. *Biochemistry.* 1997;36(39):11828-36.
5. Augusto V, Padovani C, Eduardo G, and Campos R. Skeletal muscle fiber types in C57BL6J mice. *J morphol Sci.* 2004;21(2):89-94.
